# TRF2 promotes dynamic and stepwise looping of POT1 bound telomeric overhang

**DOI:** 10.1093/nar/gkab1123

**Published:** 2021-11-25

**Authors:** Tapas Paul, Wilson Liou, Xinyi Cai, Patricia L Opresko, Sua Myong

**Affiliations:** Department of Biophysics, Johns Hopkins University, Baltimore, MD 21218, USA; Department of Biophysics, Johns Hopkins University, Baltimore, MD 21218, USA; Department of Biophysics, Johns Hopkins University, Baltimore, MD 21218, USA; Department of Environmental and Occupational Health, University of Pittsburgh, Hillman Cancer Center, 5117 Centre Avenue, Suite 2.6a, Pittsburgh, PA 15213, USA; Department of Biophysics, Johns Hopkins University, Baltimore, MD 21218, USA; Physics Frontier Center (Center for Physics of Living Cells), University of Illinois, 1110 W. Green St., Urbana, IL 61801, USA

## Abstract

Human telomeres are protected by shelterin proteins, but how telomeres maintain a dynamic structure remains elusive. Here, we report an unexpected activity of POT1 in imparting conformational dynamics of the telomere overhang, even at a monomer level. Strikingly, such POT1-induced overhang dynamics is greatly enhanced when TRF2 engages with the telomere duplex. Interestingly, TRF2, but not TRF2ΔB, recruits POT1-bound overhangs to the telomere ds/ss junction and induces a discrete stepwise movement up and down the axis of telomere duplex. The same steps are observed regardless of the length of the POT1-bound overhang, suggesting a tightly regulated conformational dynamic coordinated by TRF2 and POT1. TPP1 and TIN2 which physically connect POT1 and TRF2 act to generate a smooth movement along the axis of the telomere duplex. Our results suggest a plausible mechanism wherein telomeres maintain a dynamic structure orchestrated by shelterin.

## INTRODUCTION

Eukaryotic chromosomal ends are capped by DNA-protein complexes called telomeres, which can prevent chromosomal degradation, end-to-end fusions, and are essential for genome stability (1–3). Human telomeric DNA is composed of 2–20 kb of duplexed tandem TTAGGG repeats followed by 75–300 nucleotides of the same TTAGGG repeats at the 3′ overhang which can fold into a G-quadruplex (G4) structure (4–7). Telomeric DNA is associated with shelterin proteins including TRF1, TRF2, RAP1, TIN2, TPP1 and POT1 which protect the chromosomal ends via physical association (1,8–10). Among the six shelterin proteins, TRF1 and TRF2 directly recognize and bind to the telomeric duplex, whereas POT1 binds to telomeric overhangs (1,9). TRF1, TRF2 and POT1 bind to telomeric DNA independently without rendering cooperativity to each other (1,9). TIN2 interacts with both TRF1 and TRF2, and TPP1 forms a heterodimer with POT1 enabling association with TIN2 (10–12). The shelterin complex is located along the telomere and displays a strong affinity for binding at double-/single-stranded telomeric junctions (1,9,10). Previous evidence indicates that the shelterin complex is a dynamic structure (1,9,13), consistent with the known functions of shelterin proteins, but the dynamicity of this complex has never been demonstrated in a direct manner. Telomeres and shelterin proteins allow cells to distinguish natural chromosomal ends apart from damaged DNA (2,3,14). Upon loss or disruption of the shelterin complex, telomeres associate with at least seven distinct DNA damage response pathways including p53 dependent apoptosis (9,15–17). Hence, telomere maintenance by shelterin proteins is essential for normal cell growth, and its dysregulation may have direct consequences in aging and cancer (18).

The telomeric single-stranded overhang is critical for proper telomere function (7,19,20). POT1 is the only shelterin protein that specifically binds telomeric overhangs with a nanomolar affinity and maintains telomeric integrity (21–23). A high-resolution crystal structure revealed that human POT1 contains two oligonucleotide-binding folds (OB1 and OB2) tightly engaged with 10 nucleotides (‘TTAGGGTTAG’) with exceptionally high sequence specificity (24). OB1 makes extensive contact with the first six nucleotides of this sequence whereas OB2 binds the other four nucleotides, producing a sharp 90° kink in the DNA backbone. Two POT1 molecules are expected to bind and unfold one intramolecular telomeric G4 structure (25–27). Further, multiple POT1 units associate with longer telomeric ssDNA (24,28), likely leaving ‘GG’ gaps between two successive units based on the POT1-DNA footprint. While structural and biochemical studies reveal the exquisite POT1-DNA contact in atomic resolution and well-defined stoichiometry of the complex, it remains unknown if the POT1-bound telomere overhangs are static or dynamic in nature and if POT1-telomere structures associate with the remaining telomeric duplex and shelterin components.

TRF2 exclusively binds telomeric duplex ‘TAGGGTT’ with a nanomolar affinity (9–11). The abundance of the core shelterin components is sufficient to bind all duplex TTAGGG repeats despite variable *in vivo* concentrations of each protein (9,14,29). Further evidence suggests that TRF2, but not TRF1 plays a significant role in protecting and maintaining the integrity of the telomeric structure (30). The end of the telomere is organized in a t-loop structure which requires both a TTAGGG overhang and a duplex as blunt-ended telomeres cannot form t-loops (31). TRF2 is capable of converting telomeric DNA into a t-loop structure along the telomeric dsDNA (1,9,32). Telomere looping is a common theme in telomere architecture and is observed in all stages of the cell cycle (9,31) which serves to protect chromosome ends (33). It has been proposed that TRF2 may search for partner proteins through diffusion and may stabilize the interaction with specific telomeric DNA (13,34).

Two shelterin components, POT1 and TRF2 are critical for overall telomere function such as protecting telomeres from ATM and ATR DNA damage response (14). Both proteins maintain telomere integrity (35), regulate telomere length (36) and participate in telomere capping (35). TRF2 plays a dominant role in telomere integrity (37), yet the underlying mechanism in conjunction with other shelterin components remains unclear. Here we report that the POT1-overhang complex is inherently dynamic, making frequent contacts with the duplex DNA *in cis*. This dynamicity persists in the presence of the partner protein TPP1. Remarkably, such POT1-overhang dynamics is dramatically increased when TRF2 is engaged with the telomeric duplex. The motion entails dynamic looping and unlooping transitions which exhibit distinct stepwise movement coordinated by both proteins. We deduced the step size or the contact site to be one TTAGGG repeat along the axis of telomeric duplex. The looped state is further stabilized with increasing lengths of POT1-bound overhangs. Upon bridging interactions with TPP1 and TIN2, the discrete steps of POT1-TRF2 become spread out to smaller sub-steps, thereby generating a smoother looping-unlooping motion. We propose a plausible dynamic mechanism coordinated by POT1 and TRF2 which may facilitate the dynamic architecture of telomeres.

## MATERIALS AND METHODS

### Preparation of DNA constructs

The HPLC purified biotin and Cy3/Cy5 labeled oligonucleotides used for immobilization and for FRET measurements, respectively, were purchased from IDT (tabulated in Table S1). Each of the partial duplex DNA constructs (10 μM) were prepared by mixing a biotin-conjugated DNA strand with its complementary strand at a molar ratio of 1:1.2 (biotinylated : non-biotinylated). DNA strands were annealed in T50 buffer (10 mM Tris–HCl, pH 7.5 and 50 mM NaCl) in a thermocycler by heating to 95°C for 2 min, then gradually cooling at the rate of 2°C/min until 40°C is reached, followed by further 5°C/min cooling until 4°C. Telomeric duplexes (i.e. two and four repeat of TTAGGG duplex with overhangs) were annealed in 10 mM Tris–HCl, pH 7.5 and 5 mM MgCl_2_ containing buffer following the same protocol as described above. Milli-Q water was used to prepare all buffers and then filtered through 0.22 μm membrane filters.

### Protein purification

Recombinant human POT1 protein was expressed in a baculovirus/insect cell system and was purified as previously described (24,38). The hexahistidine sumo-tagged TPP1 (amino acids 89–334) was expressed in *Escherichia coli* cells and purified as previously described (39). TRF2 expression plasmids were transformed into BL21 (DE3) competent *E. coli* cells (NEB). Cells were cultured at 37°C until OD_600_ reached 0.4, with subsequent TRF2 expression via 50 μM IPTG induction. Proteins were expressed at 25°C for 5 h. Harvested cell pellets were lysed by sonication in 25 ml of lysis buffer (50 mM Tris–HCl, 0.6 M NaCl, 10% glycerol, 1% Tween 20, 3 mM β-mercaptoethanol, 1% NP-40, protease inhibitor tablet and 1 mM PMSF, pH 7), followed by centrifugation at 20 000 × g for 1 h at 4°C. TRF2 was purified using GST-Affinity columns. The supernatant was passed through a GSTrap™ HP column (GE), using an AKTA pure 25 M FPLC system (GE), in buffer (50 mM sodium phosphate, 100 mM NaCl and 5 mM β-mercaptoethanol, pH 7). Proteins were eluted with elution buffer (50 mM sodium phosphate, 100 mM NaCl, 5 mM β-mercaptoethanol and 25 mM reduced glutathione, pH 7). Fractions that contained TRF2 were pooled and stored in 5–10% glycerol at −80°C. SDS PAGE gel was subsequently performed and used to confirm protein purity (Supplementary Figure S1). TRF2 without GST tags produced the same result as the TRF2-GST in both 4R and 8R smFRET experiments, confirming that the TRF2-mediated enhancement of FRET fluctuations does not arise from the GST tag alone (Supplementary Figure S2).

### POT1 protein labeling

POT1 was non-specifically labeled using Cy3 NHS esters. Initially, POT1 and Cy3 NHS were mixed together in 100 mM NaHCO_3_, pH 8.5 buffer and incubated for 30 minutes at room temperature. Meanwhile, Zeba columns were prepared according to the manufacturer's instructions by spinning 300 ul of the desired buffer three times at 1500 × g for 2 min in an Eppendorf microcentrifuge. Excess dye was removed through the equilibrated Zeba column. The labeling efficiency (∼72%) was then calculated using Nanodrop spectrophotometer.

### Slide surface preparation

For all single-molecule experiments, passivated PEG slides were used to avoid any non-specific interactions of excess DNA or protein. Generally, pre-drilled quartz slides and glass coverslips are thoroughly washed with methanol, acetone, and etched by sonication in 1 M potassium hydroxide. Then, slides were burned for 2–3 min, and coverslips were quickly sterilized by passing through a flame 4–5 times to remove all sources of fluorescence. Subsequently, the slides and coverslips were coated with aminosilane for 30–45 min, then treated with a mixture of 98% mPEG (m-PEG-5000, Laysan Bio, Inc.) and 2% biotin PEG (biotin-PEG-5000, Laysan Bio, Inc.) over night. Slides and coverslips were then washed and dried using nitrogen gas and stored in −20°C for future experiments. Finally, the microfluidic sample chamber was created between the plasma-cleaned slide and the coverslip coated with PEG and biotin-PEG (40).

### Single-molecule FRET and PIFE measurements

A custom built prism-type total internal reflection (PTIR) inverted fluorescence microscope (Olympus IX 71) was used for single-molecule FRET (smFRET) measurements as described previously (41–45). Freshly annealed stocks of partial duplex DNA labeled with biotin, Cy3, and Cy5 were diluted to 15–20 pM in buffer (10 mM Tris–HCl, pH 7.5 and 100 mM NaCl). The diluted DNA was immobilized on the PEG-passivated surface via biotin–neutravidin (50 μg/ml) linkage, and unbound molecules are washed and removed using the same buffer. All smFRET measurements were carried out in imaging buffer (10 mM Tris–HCl, pH 7.5, 100 mM NaCl, 10% glycerol with an oxygen scavenging system (10 mM trolox, 0.5% glucose, 1 mg/ml glucose oxidase and 4 μg/ml catalase)) to improve dye stability and prevent fluorescent blinking. All smFRET assays were performed at room temperature (∼23°C ± 2°C). Similar procedures were followed for real-time PIFE (protein induced fluorescence enhancement) measurements using either green or red laser. All single molecule measurements which produced FRET histograms, single molecule traces, dwell time analysis, heatmap and violin plots presented in the manuscript were repeated at least three times on different days with each experiment yielding over 50,000 single molecule traces (40).

### Data acquisition

The evanescent field was generated through PTIR using a solid state of either 532 or 634 nm diode laser (Compass 315M, Coherent) to excite the fluorophores (Cy3 or Cy5) at the sample chamber. The fluorescence from the fluorophores (Cy3 (donor) and Cy5 (acceptor)) were simultaneously collected using a water immersion objective. A dichroic mirror (cut off = 630 nm) was used to separate and project the emission onto an EMCCD camera (Andor). Data was recorded with 100 ms frame integration time, processed with an IDL script (http://www.exelisvis.co.uk/ProductsServices/IDL.aspx), and finally analyzed by Matlab scripts (https://www.mathworks.com/).

### smFRET data analysis

FRET histograms were generated from more than 4000 molecules (21 frames of 20 short movies) collected from different imaging surfaces. To exclude donor only molecules from the histogram at the low FRET region, green and red lasers were sequentially used to excite Cy3 and Cy5 respectively (10 frames for Cy3, 1 frame dark and 10 frames for Cy5). Additionally, the donor-leakage was corrected based on the FRET values of donor-only molecules. The histograms were normalized and fitted to Gaussian distributions in Origin 2018 (https://www.originlab.com/) with unrestrained peak center position. A Matlab code was used to measure the dwell time which was then single-exponentially fitted in Origin 2018 to extract decay times. The FRET heatmaps were generated by an in-house MATLAB script overlaying >100 traces. HaMMy fitting of single-molecule time traces and transition density plots (TDP) were generated by using free software (https://cplc.illinois.edu/software/). To obtain the number of discrete FRET states of our given system, we HaMMy-fitted the dynamic smFRET traces of all constructs. We applied more than the number of expected FRET states, i.e. six or eight states for HaMMy fitting (see Supplementary Figure S3). HaMMy determined the most likely combination of FRET states and the corresponding transition rates such as state-to-state transition probabilities (46,47). The transition density plot (TDP) was generated by using HaMMy-fitted numbers of each transition, irrespective of the given number of states.

### smFRET real-time experiment

The smFRET real-time POT1 binding to G4/4R and TRF2 binding to telomeric duplex assays were carried out with a micro-fluidic imaging flow chamber. A small piece of plastic was placed above the pre-drilled holes at one end of the chamber to serve as a reservoir for buffer flow, while pre-drilled holes at the opposite end of the chamber were connected to a silicone tube with a syringe pump (Harvard Apparatus, MA). Either POT1 or TRF2 suspended in imaging buffer was loaded into the plastic reservoir. Real-time FRET images were collected by passing solution through the imaging chamber at a flow rate of 20 μl/s. The smFRET time trajectories were analyzed using Matlab scripts. Using the individual single-molecule real-time flow traces, FRET flow heatmaps were generated. POT1 binding kinetics were calculated from the moment of flow to the moment of first irreversible FRET decline. In each case more than 100 molecules were analyzed.

### Heatmap generation and violin plot

Heatmap histograms were generated by combining dynamic traces using Origin 2018. Flow heatmaps were generated by combining and analyzing traces using Matlab scripts. We measured the fraction of time each molecule spends in the dynamic state and plotted using a violin plot MATLAB script. Violin plot MATLAB code was available online through MATLAB file exchange (https://www.mathworks.com/matlabcentral/fileexchange/45134-violin-plot).

## RESULTS

### POT1 remains stably bound to telomere overhangs

**Figure 1.**
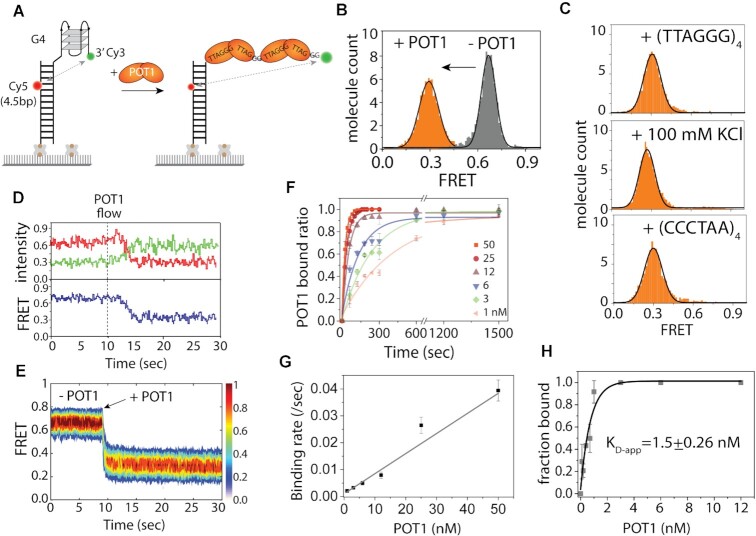
POT1 is stably bound to telomeric G4/4R. (**A**) Schematic smFRET model of before and after POT1 (two orange lobes represent OB1 and OB2 domains of a single POT1 molecule) binding to telomeric G4/4R DNA (Top4.5 construct). (**B**) The FRET histograms of 4R before and after POT1 (25 nM) binding. In y-axis, molecule count means the normalized number of molecules. (**C**) FRET histograms after 30 min incubation with 4R only (top), 100 mM KCl (center) and C4 (bottom) to the POT1 bound G4. (**D**) The representative real-time smFRET trace of POT1 binding to 4R (protein flow at ∼10 s). (**E**) The heatmap (*n* > 100), generated by synchronizing the POT1 bound state. (**F**) Single-exponential fitting of POT1 bound fraction to 4R overhang at different POT1 concentrations. (**G**) Linearly fitted binding rate represents the corresponding POT1 binding rate to 4R overhang at different concentration. (**H**) Determination of the apparent dissociation constant (*K*_D-app_) of POT1 to 4R/G4.

POT1 is a unique protein in that it binds exclusively to telomere overhangs with high affinity and sequence specificity in a well-defined structural organization (24). Previously, we showed that POT1 domains associate with human telomeric overhangs (TTAGGG)_4_ sequentially, one oligonucleotide/oligosaccharide binding (OB) fold at a time (25). Here, we asked if the POT1 bound telomere overhang remains stably engaged without dissociation or conformational dynamics over time. For this goal, we varied FRET dye positions to assess the status of the POT1-overhang complex more accurately. The substrate contained telomeric ssDNA composed of four TTAGGG repeats, or 4R (forms one complete G4) and an 18 bp duplex for immobilization via biotin-NeutrAvidin to a slide (25). We use ‘G4’ and ‘4R’ to denote G-quadruplex and four repeats of TTAGGG, respectively. The construct consists of a donor (Cy3) dye at the 3’ end with the acceptor (Cy5) dye labeled in between the fourth and fifth base pair away from the double-/single-strand (ds/ss) junction, hence termed Top4.5 (Figure 1A). FRET histograms were built by collecting FRET values from >4000 molecules in 20 different fields of view. The 4R sequence exhibits a mid-FRET peak ∼0.65 due to the folded G4 and the 4.5 bp spacing from the ds/ss junction (Figure 1B). After the addition of POT1 (25 nM), the mid-FRET peak completely shifted to low FRET at ∼0.3, indicating complete binding of POT1 (Figure 1B). The experiments were carried out in NaCl (100 mM) because the KCl (100mM) condition prevents efficient POT1 binding of telomeric G4 overhang (24,25,27). To test how stable the POT1-overhang complex is, we performed a competition assay by applying ten-fold molar excess of unlabelled 4R (no 18 bp), C4 (complementary sequence of [TTAGGG]_4_), and 100 mM KCl (G4 stabilizing condition, POT1 does not bind overhangs at 100 mM KCl) (27,48) to the POT1 bound overhang. After 30 min of incubation, the FRET histogram remained unchanged at FRET∼0.3 in every case (Figure 1C), clearly indicating the exceptional stability of POT1-overhang complex.

Real-time POT1 binding to 4R/G4 revealed a clear mid FRET to low FRET transition immediately after the POT1 addition (Figure 1D). More than 100 single-molecule traces were combined to generate a heatmap in which each trace was synchronized at the moment of FRET decrease (Figure 1E). This rapid transition from free G4 (∼0.65 FRET) to the POT1 bound state (∼0.3 FRET) indicates a rapid POT1 binding to the telomere overhang. Furthermore, we obtained smFRET traces that exhibit one (∼52%), two (∼33%), three (∼<10%) and four (∼<10%) steps of POT1 binding (Supplementary Figure S4). The stepwise FRET decrease corresponds to the OB folds of POT1 binding sequentially, which is consistent with our previous finding (25). For kinetic analysis, varying concentrations of POT1 from 0.1 nM to 50 nM was applied to FRET-labeled G4. The binding time was calculated based on the dwell time between POT1 addition and the subsequent FRET decline from the smFRET traces (for [POT1] > 10 nM). We note that we did not select any molecules to use for binding rate since in this concentration, all DNA molecules underwent binding of POT1, exhibiting the same pattern of FRET decrease. For low concentrations, due to the slow binding, we used the bound fraction calculated from FRET histograms taken over time (for [POT1] < 10 nM). The binding rates were determined by exponentially fitting the bound fractions with respect to time (Figure 1F). The rate increases linearly as a function of POT1 concentration (Figure 1G). Since the protein remains bound without dissociating, we calculated the apparent equilibrium dissociation constant (*K*_D-app_) to be 1.5 ± 0.3 nM (Figure 1H), confirming an extremely high affinity of POT1 to telomere overhang.

### POT1-bound telomeric overhangs display dynamic motion

**Figure 2.**
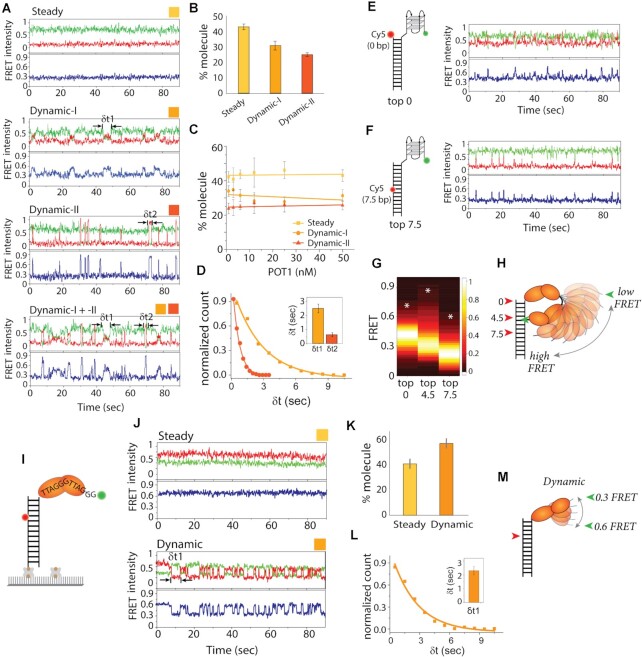
POT1 bound telomeric overhang shows dynamics. **(A)** The representative smFRET traces of POT1 bound to telomeric 4R after wash of free protein show steady (FRET ∼0.3, top), Dynamic-I (FRET ∼0.2–0.6, second from top), and Dynamic-II (FRET ∼0.2–0.9, bottom two) traces. (B, C) Quantification of molecular behavior of POT1 bound G4 (steady versus two types of dynamic) at 25 nM after wash of free protein (**B**) and the protein concentrations ranging from 50 to 1 nM before wash of free protein (**C**). (**D**) Dwell time of dynamic FRET traces, δ*t*1 for Dynamic-I and δ*t*2 for Dynamic–II respectively. (E, F) Model cartoon of Top0 (**E**) and Top7.5 (**F**) 4R construct, based on the acceptor dye position from the top of the duplex and beside the representative POT1 bound smFRET traces respectively. (**G**) FRET heatmap histogram of three constructs generated from the dynamic traces (keeping the bin size 0.2). White asterisk denotes level of highest FRET values observed in traces. (**H**) Schematic dynamic model of POT1 bound telomeric G4/4R overhang. (**I**) Schematic smFRET model of monomer POT1 bound to telomere overhang. (**J**) The representative smFRET steady (top) and dynamic (bottom) traces of POT1 bound to telomeric 2R overhang. (**K**) Quantification of molecular behavior of POT1 bound 2R (steady vs dynamic). (**L**) Dwell time (δ*t*1) of dynamic FRET traces of 2R overhang. (**M**) Schematic dynamic model of POT1 bound telomeric 2R overhang.

The above observation of the unchanging FRET histogram even after three competing conditions demonstrates that two POT1 monomers are stably engaged with the telomeric G4 overhang. To check if such a stable interaction is maintained in a static state over time, we looked through single-molecule traces taken before and after washing off unbound POT1. In both conditions, while some traces showed a steady FRET at ∼0.3 which corresponds to the steady POT1 bound state (Figure 2A), other traces displayed FRET fluctuations, indicating a dynamic state of the POT1-overhang complex. Among the dynamic traces, we found two different patterns categorized as Dynamic-I and –II based on the FRET fluctuation pattern. Dynamic-I includes traces that show slower FRET fluctuations oscillating between ∼0.2 and 0.6 whereas Dynamic-II contains sharp FRET peaks that reaches above ∼0.6 FRET, often rising up to 0.9 which is higher than the G4-only FRET (Figure 2A, more traces, in Supplementary Figure S5A). Dynamic-I + II refer to traces that had a mixture of both dynamic patterns. These dynamics spikes occur stochastically, varying in frequency. The dynamics are observed immediately after POT1 binding as shown in the real-time smFRET traces (Supplementary Figure S5B). This POT1-induced overhang dynamic is not due to nonspecific binding to surface (Supplementary Figure S6) nor from the multimerization of POT1 protein as POT1 remains monomeric in solution (49). Based on the criteria stated above, we categorized over 1000 smFRET traces into steady, Dynamic-I, or Dynamic–II traces. Approximately, ∼40–45% traces were steady while ∼30–35% were Dynamic-I and ∼25–30% were Dynamic-II (Figure 2B). To check if the dynamics were concentration-dependent, we titrated the protein to concentrations ranging from 1 nM to 50 nM. Remarkably, the distribution of steady vs. dynamic pattern remained the same in all POT1 concentrations both before and after the wash of free protein (Figure 2C). To test if the stable FRET state arises from one POT1 bound to a central registry, we prepared (TTAGGG)_2_ surrounded by T_6_ such that there is only one POT1 binding site in the middle within the same length of ssDNA to G4. POT1 binding to this construct led to 0.45 FRET state which is different from the 0.3 FRET state that emerges from POT1 bound to G4 (Figure S7), ruling out the possibility of one POT1 bound to a central registry. Furthermore, to test the importance of having fully correct TTAGGG repeat sequences for generating overhang dynamics, we mutated the first guanine in the most 3′ side of telomeric overhang to C, T and A. Previously, we showed that POT1 binding to telomeric overhang is not affected by this single nucleotide substitution (27,48). In agreement, the result shows that the same pattern and level of FRET fluctuation is exhibited in the singly mutated overhang sequence (Supplementary Figure S8). Taken together with the stable association of POT1 to the telomere overhang (Figure 1C), this clearly indicates that the POT1-induced conformational dynamics arise from the stably bound configuration rather than binding and unbinding events. Next, we analyzed the dwell time representing the frequency of FRET fluctuation and found that Dynamic-I (δ*t*1 = 2.51 ± 0.31 s) is ∼4-fold slower than the Dynamic-II (δ*t*2 = 0.63 ± 0.14 s), consistent with the long- and short-lived excursions seen in Dynamic-I and -II, respectively (Figure 2D).

To further probe the POT1-induced conformational dynamics at the telomeric overhang, we modified the FRET DNA construct by repositioning the acceptor dye to the top of the duplex (Top0) in one construct and in-between the seventh and eighth base pairs (Top7.5) in another (Figure 2E, F). POT1 was applied to both constructs and the smFRET traces were examined after washing out free protein. Both constructs showed dynamic FRET albeit with less pronounced FRET change compared to the Top4.5 construct (Figure 2E, F). While the smFRET traces for Top0 (FRET ∼0.4, POT1 bound state) showed FRET decreases to 0.2 and increases to ∼0.7 (more traces in Supplementary Figure S9), the Top7.5 traces (FRET ∼0.2, POT1 bound state) displayed only FRET increases to ∼0.5. To compare the overall FRET fluctuation range exhibited in the three DNA constructs, we generated a heatmap depicting the FRET fluctuation dynamics collected from over 200 smFRET traces in each condition (Figure 2G). The major FRET level (brightest band) is different for the three constructs due to the different dye positions despite the same POT1-induced state. Interestingly, the Top4.5 displays the widest span of FRET values (0.15–0.9) compared to the other two. Top0 and Top7.5 each shows FRET range between 0.2–0.7 and 0.1–0.6, respectively. Such differences in FRET range serve as a proxy for the path taken by the mobile POT1-overhang complex with respect to the duplex DNA.

Most human telomere ends with sequence TTAG, therefore we used 3R TTAG where two POT1 proteins can bind in a similar manner to 4R telomere overhangs. The molecular behavior, quantification of steady versus dynamic states, and the range of high FRET dynamic between 4R and 3R TTAG telomere overhangs are comparable (Supplementary Figure S10). Taken together, the POT1-bound telomeric 4R overhang is not a static structure, but a highly dynamic complex. The unexpected high FRET of 0.9 shown with the Top4.5 construct indicates that the 3′ end of POT1-overhang approaches the 4–5th base pair of duplex to an extremely close proximity, approximately within 3 nm. This result suggests a transient and dynamic conformation formed by POT1 bound telomere overhang as depicted in Figure 2H.

### The monomer POT1-bound telomeric overhang displays a dynamic state

Next, we asked if the dynamic conformational change arises from two units of bound POT1 since the DNA structure predicts two guanines spaced between the two tandem binding of POT1 molecules (24), which may serve as a pivot point for the observed motion. To test this, we examined a monomer POT1 bound condition on a shortened overhang construct composed of two repeats of TTAGGG (2R) with the same Top4.5 acceptor dye arrangement which should only allow for one POT1 binding (Figure 2I and Supplementary Figure S11A). Due to the short length of the overhang, the DNA yields ∼0.8 FRET which immediately shifted to ∼0.6 FRET upon POT1 addition (Supplementary Figure S11B). The *K*_D-app_ of POT1 for 2R was 1.4 ± 0.2 nM, which is 5-fold tighter than previously reported (24) and comparable to the affinity toward 4R overhang (Supplementary Figure S11C). Such a difference in *K*_D-app_ likely arises from the different experimental approach used in our measurement. For example, the 50–100 pM concentration of DNA used in our single molecule assay may be one to two magnitude lower than other conventional methods, thus contributing to the difference. The smFRET traces showed a mixture of a constant 0.6 FRET corresponding to the steady binding state, and oscillating FRET states between 0.6 and 0.3 as the dynamic state (Figure 2J, more traces in Supplementary Figure S12). Considering the 0.6 FRET as the POT1 monomer bound state and 0.3 as lower than the 0.5 FRET observed in unfolded poly dT12 substrates (same length as 2R) (50), fluctuation between 0.6 and 0.3 indicate that the 3′ telomeric end displays excursions to a fully extended conformation (depicted in Figure 2M). As done for 4R, we quantified the fraction of steady vs. dynamic traces and found ∼40% steady and ∼60% dynamic behavior, which is similar to the case of two POT1 molecules bound to the 4R overhang (Figure 2K). The dwell time collected from the dynamic FRET transitions (δ*t*1 = 2.31 ± 0.29 sec) was similar to the Dynamic-I obtained for 4R overhang (δ*t*1 = 2.51 ± 0.31 s) (Figure 2L), likely suggesting that Dynamic-I represented a monomer mediated conformational change while Dynamic-II arose from the two monomer bound state. Therefore, it is possible that Dynamic-I arises from the flexible junction between OB1 and OB2 domain whereas Dynamic-II originates from rotational movement pivoting around the two guanine linker between the two POT1 units. Altogether, the POT1-overhang induced dynamics occurs even at the monomer level, likely entailing full straightening of the 2R overhang which may be accompanied by stretching out or unbending of OB1 and OB2 domains within a monomer (Figure 2M).

### TRF2 enhances POT1 induced overhang dynamics

**Figure 3.**
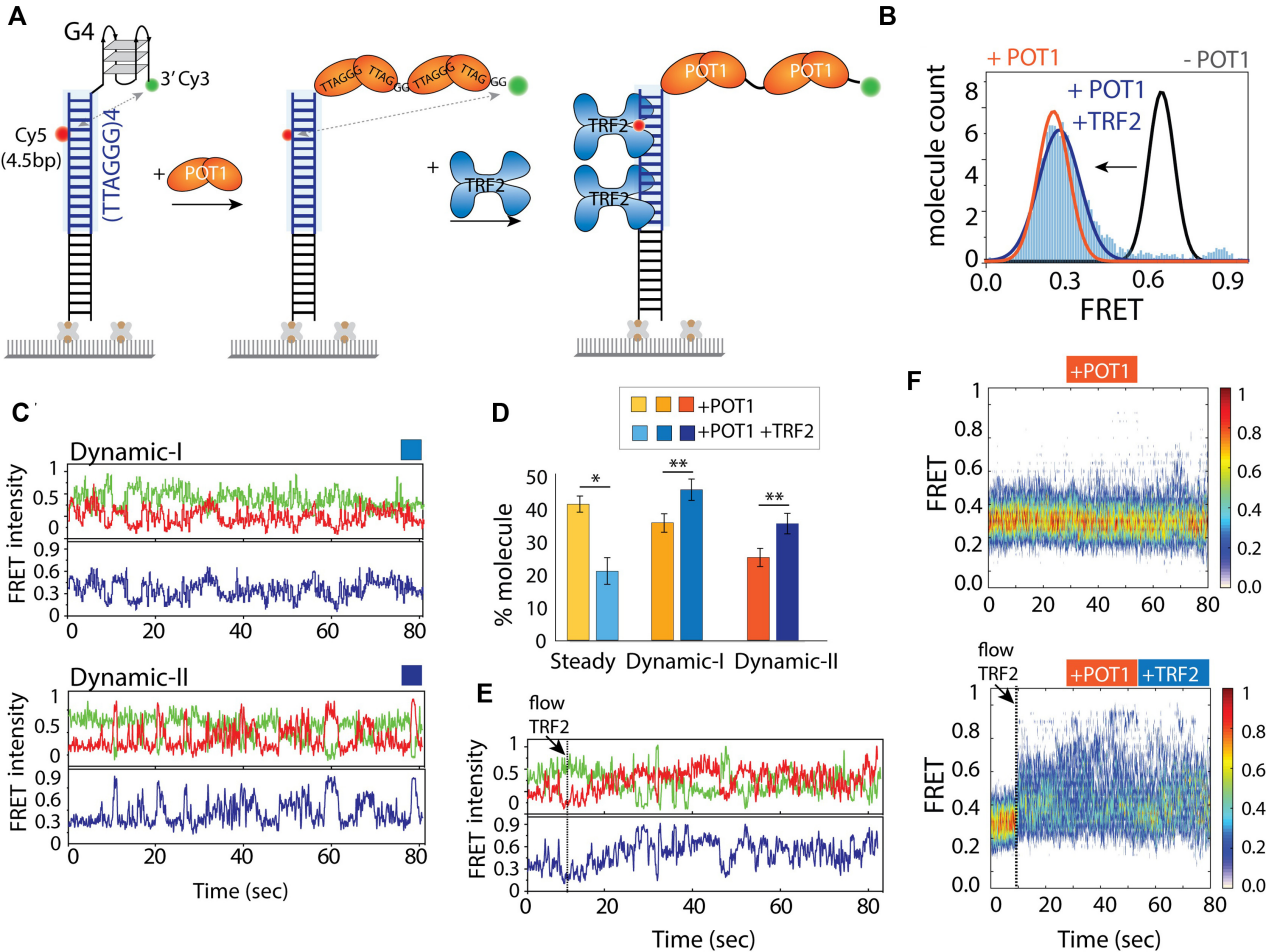
Telomeric duplex bound TRF2 enhances POT1-overhang dynamics. (**A**) Schematic model of telomeric duplex (four repeats of TTAGGG tract) with G4 overhang sequentially binding POT1 at the overhang followed by TRF2 at the duplex. The basic domain of TRF2 can bind to ss-ds junction, for simplicity isn’t shown here. (**B**) The FRET histograms of telomeric duplex with G4 (∼0.65 FRET, black solid line), POT1 bound condition (∼0.3 FRET, orange solid line), and POT1, TRF2 together (∼0.3 FRET, solid blue filled). (**C**) POT1 and TRF2 bound representative smFRET traces of Dynamic-I (top) and Dynamic-II (bottom) states. (**D**) Quantification of molecular behavior (steady vs two types of dynamics) of 4R bound POT1 in presence and absence of TRF2. All statistics for this figure are calculated using a two-tailed two-sample Student's *t* test (*P < 0.05; **P < 0.01). (**E**) Representative smFRET trace of real time TRF2 binding at telomeric duplex (flow at ∼10 s) contain POT1 pre-bound overhang. (**F**) The heatmap (*n* > 100) of POT1 bound state in absence (top) and presence (bottom) of TRF2 (flow at ∼10 s).

Results thus far suggest that the POT1 bound telomeric overhang undergoes dynamic conformational change at both monomer and dimer levels. Next, we asked if such behavior changes upon TRF2 binding to telomeric duplex *in cis* as TRF2 is involved in telomere end protection (32). To test the role of TRF2, we extended the dsDNA by inserting four TTAGGG repeats of telomeric duplex to the Top4.5 construct (Figure 3A). First, we checked if the telomeric duplex has any impact on POT1 induced dynamics. We performed the identical POT1 binding assay and found that the FRET pattern is highly similar to the non-telomeric duplex with 4R (Figure 3B), including nearly identical distribution and kinetics of Dynamic-I and –II (Supplementary Figure S13). Hence, we confirm that the telomeric duplex doesn’t modulate POT1 induced telomere overhang dynamics.

The shelterin component, TRF2 binds telomeric duplexes as a homodimer with high sequence specificity of ‘TAGGGTT’ (9,10). Thus, one TRF2 homodimer occupies two TTAGGG telomeric duplex repeats, matching the length requirement of one POT1 binding on the overhang. When TRF2 (25 nM) was added to the DNA with a telomeric duplex (Figure 3A), the FRET peak at 0.65 remained the same, making it impossible to check for binding (Figure 3B). The lack of FRET change may be due to either no binding or binding without altering the FRET value. To test the TRF2 binding more directly, we performed protein-induced fluorescence enhancement (PIFE) (51,52) experiments on both Cy3 and Cy5 dyes, which showed clear increases in Cy3 and Cy5 intensity signifying TRF2 engagement (binding rate ∼ 0.13 ± 0.2 s^–1^) (Supplementary Figure S14). PIFE experiments were conducted independent of FRET, i.e. PIFE signal change was the only readout. Likewise, aforementioned FRET experiments were conducted in a PIFE-independent manner. Due to the ratiometric analysis of FRET, we do not have to consider the contribution of PIFE in most cases. Therefore, TRF2 binds the telomeric duplex without changing the G4 conformation, consistent with previous findings (10,11). This result also indicates that TRF2 alone does not change the conformation of folded G4.

Next, we tested the POT1-overhang dynamics in the presence of TRF2 bound to the telomeric duplex. POT1 and TRF2 were added sequentially to our smFRET construct. When TRF2 was added to the POT1 bound state, the FRET peak at ∼0.3 (POT1 bound state) appeared with a slightly broader width, likely indicating the TRF2 impact on the POT1 bound overhang (Figure 3B). Strikingly, the smFRET traces revealed remarkably increased occurrence of both Dynamic –I and -II states with TRF2 (Figure 3C, D). The trace classification yielded ∼20% steady, ∼45% Dynamic-I, and ∼35% Dynamic-II, altogether comprising ∼80% dynamic motion, compared to 60% in POT1 alone without TRF2 (Figure 3D). The enhanced frequency of dynamics strongly indicates the influence of TRF2 in reinforcing the POT1 induced overhang dynamics with the duplex. To assess the time for TRF2 to impart its modulation of POT1-bound overhang dynamics, we examined real-time smFRET traces which were collected before TRF2 addition to ∼3 min after TRF2 addition. Overall, we observed a rapid FRET increase immediately after TRF2 addition, followed by robust FRET fluctuations as seen in a majority of the traces (Figure 3E). To demonstrate this effect collectively, we generated a heatmap by combining real-time dynamic traces (>50) (Figure 3F, bottom). Such an effect was not observed in the absence of TRF2 (Figure 3F, top) or on non-telomeric duplexes (Supplementary Figure S15). This suggests that TRF2 does not interact with non-telomeric duplexes *in trans*, or directly with POT1 in the absence of telomeric duplex. Therefore, TRF2 promotes POT1 induced overhang dynamics only *in cis*, in the context of the TRF2-bound telomeric duplex.

To directly probe POT1-induced overhang dynamics and the impact of TRF2, we labeled POT1 with Cy3 and performed the same experiment on Cy5-labeled DNA which is otherwise unchanged (Supplementary Figure S16A). Interestingly, Cy3-labeled POT1 bound to the telomeric overhang showed dynamics that resembles the case with two dyes on DNA, reflecting that POT1 is indeed generating conformational dynamics. In addition, such dynamic motion was significantly enhanced in the presence of TRF2, again consistent with the previous result (Supplementary Figure S16B). Due to the non-specific labeling of POT1 and possibility of having two labeled POT1 on one overhang, the traces are not as uniform as before. Regardless, the heat map generated by combining dynamic FRET traces and the population density plot in the form of a violin plot both reflect the increased FRET fluctuation and higher amplitude of FRET change in the presence of TRF2 (Supplementary Figure S16C, D).

### TRF2-POT1 induced dynamics is independent of overhang length

**Figure 4.**
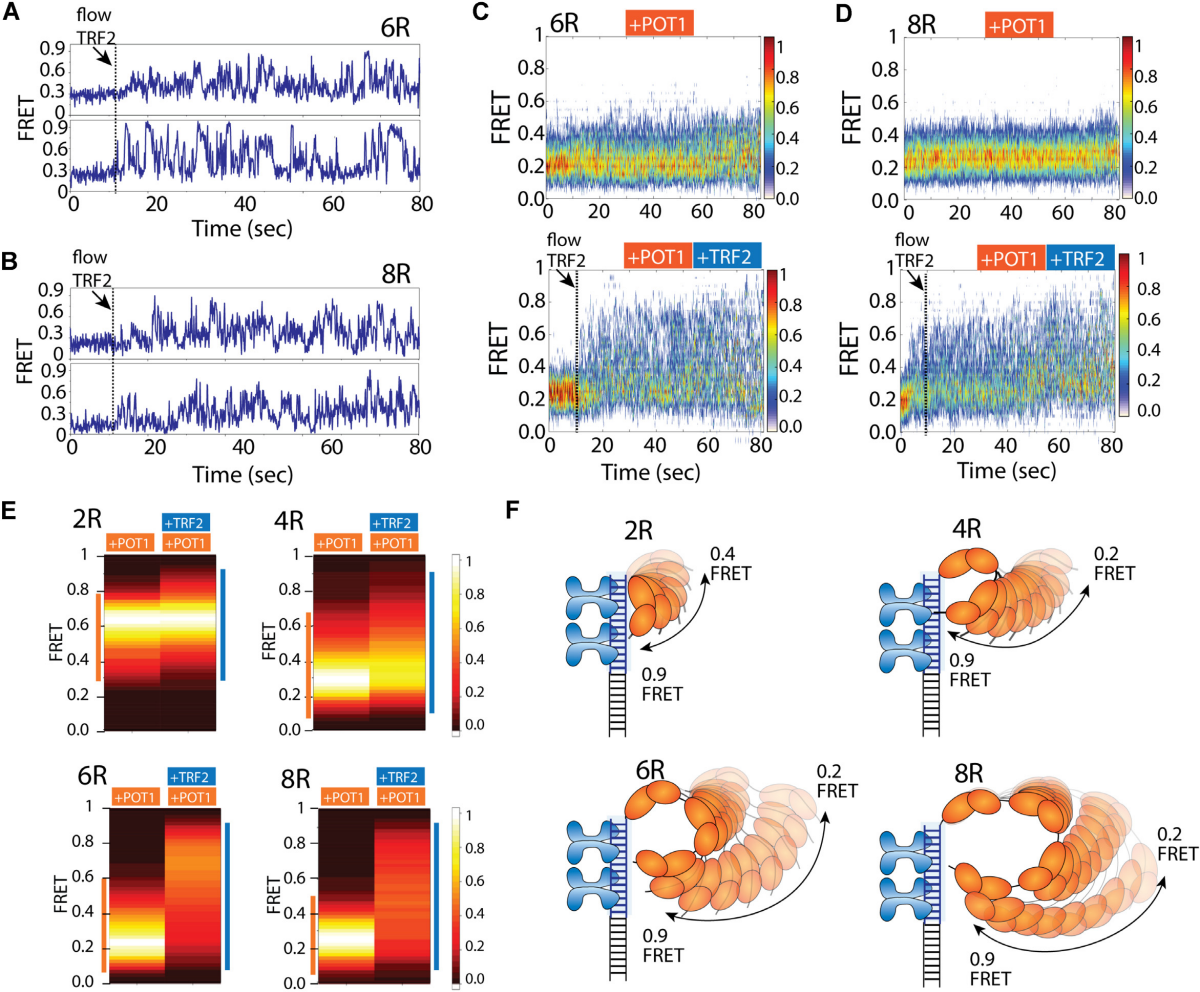
Enhanced dynamics also observed in longer telomeric overhang. (A, B) The representative real time smFRET traces of TRF2 binding at telomeric duplex (flow at ∼10 s) to the pre-bound POT1 of 6R (**A**) and 8R (**B**) overhangs. (C, D) The heatmap (*n* > 100) of POT1 bound state in absence (top) and presence (bottom) of TRF2 (flow at ∼10 s) of 6R (**C**) and 8R (**D**) overhangs. (**E**) FRET heatmap histograms generated from the dynamic traces of POT1 only and POT1 followed by TRF2 addition to 2R, 4R, 6R and 8R overhangs containing telomeric duplex (keeping the bin size 0.2). (**F**) Proposed model of TRF2 induced POT1 overhang dynamics of the respective construct.

Based on the results above, we hypothesized that the frequent contact made between the POT1-bound overhang and duplex associated TRF2 may represent a transient, yet persistent loop forming activity. We reasoned that if such motion is coordinated by POT1 and TRF2, similar movement may persist even when the overhang length is extended. On the contrary, if the motion was due to random fluctuations, the longer overhang of 6 to 8 repeats will less likely be in the FRET-sensitive distance range. To test this, we lengthened the telomeric overhang from four TTAGGG repeats (4R) to six (6R) and eight repeats (8R) while keeping the same four repeats of telomeric duplex. POT1 binding to 6R and 8R overhangs yielded low FRET ∼0.2, consistent with the POT1 bound state (53). The smFRET traces obtained in POT1-bound state showed dynamics, but with a diminished FRET range, likely arising from the stretched conformation of long overhangs as the total ssDNA length of 6R (36 nt) and 8R (48 nt) exceed the FRET sensitive distance range (50). In contrast, when TRF2 was added to 6R and 8R pre-bound with POT1, we observed a sudden burst of FRET fluctuation which displays remarkably high FRET states up to 0.9 (Figure 4A, B). Again, the appearance of high FRET emerged immediately after TRF2 addition, signifying the role of TRF2 in triggering increased POT1-overhang dynamics. The heatmap generated by combining real-time traces (>50) clearly reveals the instantaneous rise in FRET value concomitant with TRF2 binding (Figure 4C, D). In the absence of TRF2, FRET ranges are more confined due to less molecules showing dynamics and less time spent in the dynamic state. The extremely high FRET of 0.9, despite the long length of overhang (6R, 8R) clearly indicates a completely looped configuration in which the 3′ end of the POT1 bound overhang is recruited adjacent to TRF2 near the ds/ss junction.

To compare the POT1-overhang dynamics exhibited in the absence or presence of TRF2 in all four constructs, we collected FRET values from traces with representative FRET fluctuations in both conditions and plotted the range of FRET as heatmap histograms for 2R, 4R, 6R and 8R (Figure 4E). While the FRET ranges observed in POT1 bound state vary as a function of telomeric overhang length, the TRF2-induced FRET fluctuation produced significantly greater variation in the FRET amplitude. For the shortest telomeric overhang, 2R which accommodates one POT1, the FRET range is similar with or without TRF2 due to the restricted movement in the short arm of the 12 nucleotide overhang (Figure 4E, left top). For 4R, 6R and 8R overhangs, the difference between POT1 without and with TRF2 become substantially more pronounced with the maximum difference exhibited by 8R in which the dynamic FRET range spans 0.1 to 0.9 (Figure 4E, right bottom). Taken together, POT1 bound overhangs are inherently dynamic and TRF2 bound *in cis* stimulates exceptionally dynamic loop formation by bringing the very end of 3′ overhang to the telomere ds/ss junction repeatedly and persistently regardless of the length of the POT1-bound overhang (Figure 4F).

### Dynamic FRET states involve discrete steps

**Figure 5.**
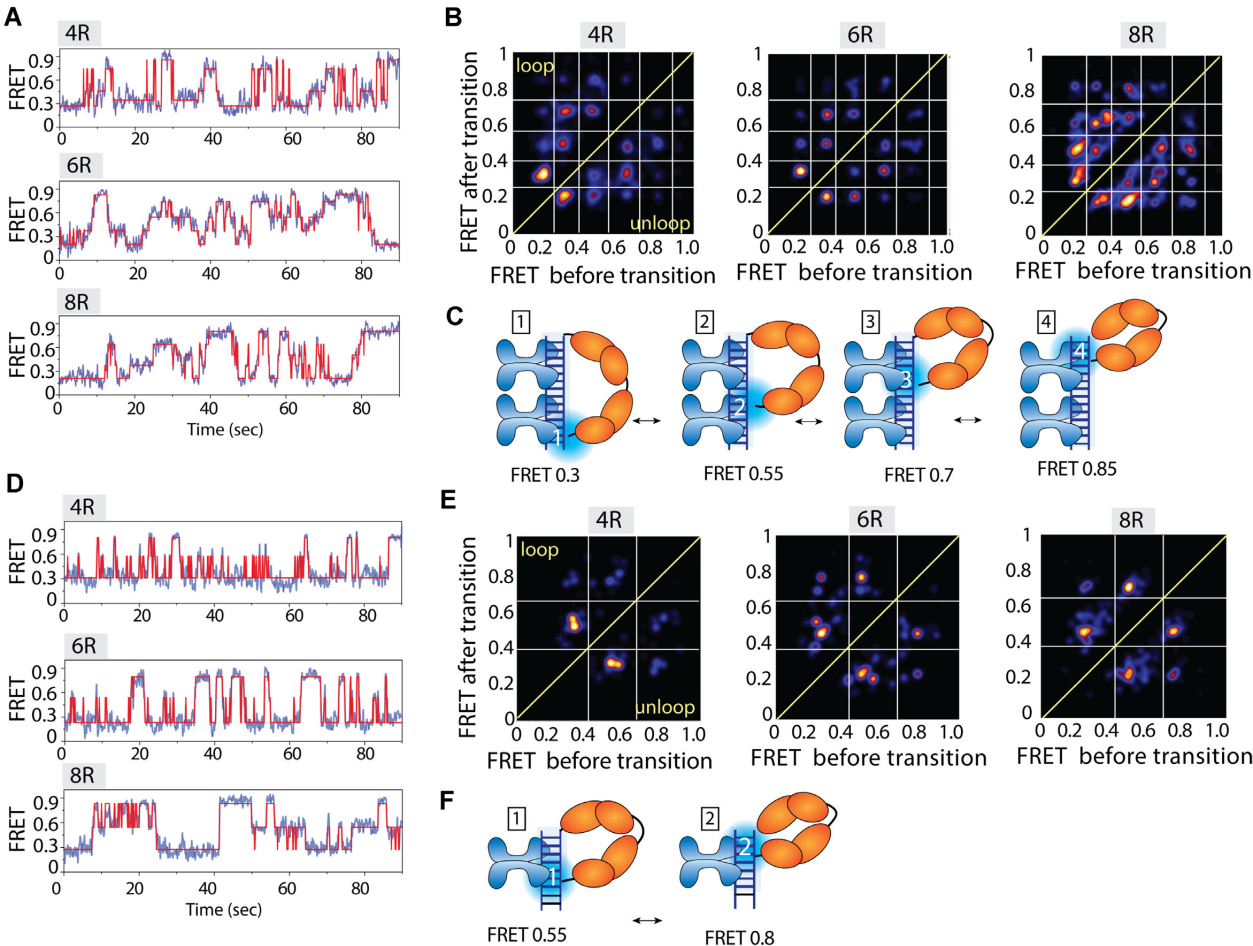
POT1 bound overhangs show discrete steps moving up and down along the TRF2 bound duplex. (**A**, **D**) Representative smFRET traces (blue) fitted by HMM (red), (**B**, **E**) transition density plot (TDP) and, (**C**, **F**) the proposed models of POT1 bound 4R, 6R and 8R telomeric overhangs moving up and down to the TRF2 bound four (A–C) and two (D–F) repeats of TTAGGG containing duplex.

Upon close examination of smFRET traces collected with POT1 and TRF2, we recognized that the FRET transitions between 0.1 and 0.9 were not smooth and continuous, but rather uneven and non-uniform (i.e there were discrete states which made the traces look jagged) (Supplementary Figure S17). To check if there are discrete steps taken along the dynamic path, we employed an unbiased approach, termed hidden Markov Model (HMM) to analyze the dynamic smFRET traces. HMM analysis is ideal for identifying statistically significant distinct FRET levels within noisy smFRET traces (46). The HMM algorithm was applied to fit smFRET traces (by applying different numbers of states, *N* = 3–8) obtained from POT1-TRF2 experiments performed with 4R, 6R and 8R (Figure 5A and Supplementary Figure S3). For each construct, more than 50 dynamic traces were HMM fitted and the number of steps with their FRET values were extracted. All results of HMM analysis were combined to generate a transition density plot (TDP) for all three constructs (54). TDP is a two-dimensional histogram plotted by taking FRET before transition on the x-axis and FRET after transition on the y-axis. Therefore, the ascending vs. descending FRET levels are populated on the upper left and lower right triangle coordinates, respectively. The interpretation is that the ascending and descending steps represent looping and unlooping transitions, respectively. As a density plot, the intensity of each spot indicates the visiting frequency at the given FRET state. The HMM analysis identified four distinct FRET states in TDP corresponding to four discrete steps of transitions embedded within the dynamic smFRET traces (Figure 5B). Interestingly, the 4R, 6R and 8R overhangs all showed the same pattern with highly similar FRET values (i.e. ∼0.35, ∼0.55, ∼0.7 and ∼0.85 FRET states) (Figure 5B). We note that some traces exhibit less than four states, yet the three, two, and one steps of transitions all converge to the same four FRET states listed above, indicating well defined positions of contact between POT1-overhang complex and TRF2-duplex. The emerging picture is that POT1 is moving up and down the axis of the TRF2 bound telomeric duplex, undergoing differently looped states in a stepwise manner. The four distinct steps likely involve four units (two sets of homodimers) of TRF2 bound to telomeric duplex (Figure 5C). Such movement is only observed when both POT1 and TRF2 are present (Supplementary Figure S18A). The individual lifetime of the different FRET state reveals the lifetime of highest FRET state (∼0.85) was the highest in 8R followed by 6R and 4R, suggesting that the most looped state is stabilized by longer POT1 bound overhangs, likely due to less tension generated in looping longer POT1-overhang complexes (Supplementary Figure S18B). Interestingly, TRF2ΔB which lacks the basic domain that contacts the C-terminal domain of POT1, was not able to induce the highest FRET state evidenced by the majority of smFRET traces displaying low to mid FRET dynamics devoid of the high FRET values of 0.7–0.9 (Supplementary Figure S19). This indicates that TRF2ΔB cannot recruit the POT1-bound 3′ end to the ds/ss junction, consistent with the known function of this basic domain in stabilizing the ds/ss three-way DNA junction (55).

To test if the number of FRET states correspond to the stoichiometry of TRF2, we reduced the valency of TRF2 to two (one homodimer) by shortening the length of the telomeric duplex to two TTAGGG repeats. Upon applying the same POT1 and TRF2 conditions, we indeed obtained two distinct FRET states (∼0.55 and ∼0.8) of dynamics from the HMM fitting of smFRET traces from 4R, 6R and 8R overhangs (Figure 5D). Subsequently, two discrete steps of transitions appeared on TDP analysis of all three constructs indicating two ascending and two descending steps of movement taken by POT1-overhangs along the axis of TRF2-duplex (Figure 5E). Hence, the discrete steps between the two proteins correspond to the distinct pairing between POT1 and TRF2 and the number of steps is proportional to the number of TRF2 units on a duplex (Figure 5C, F).

### TPP1 and TIN2 generate more dynamic states within the TRF2-POT1 complex

**Figure 6.**
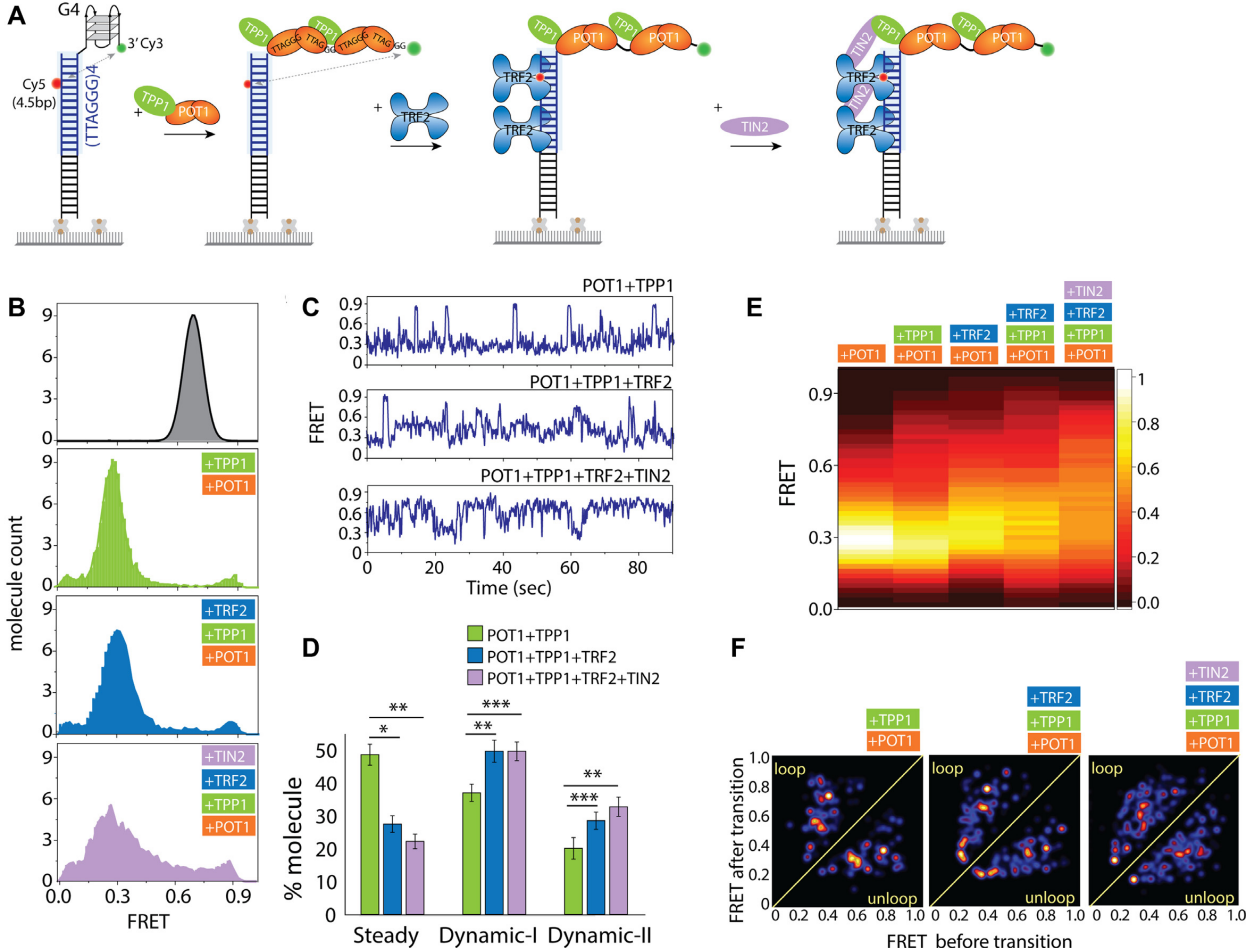
TPP1 and TIN2 generates smaller substeps within the TRF2-POT1 induced movement. (**A**) Experimental schematics of four shelterin components added in succession to telomeric duplex (four repeats of TTAGGG tract) with G4/4R overhang. First, premixed POT1–TPP1 is applied followed by TRF2 and TIN2 which interact with TRF2 and TPP1. (**B**) FRET histogram of telomeric duplex with 4R (top), with POT1-TPP1 (second from top), with TRF2 (third from top) and TIN2 bound condition (bottom). (**C**) Representative smFRET traces. (**D**) Quantification of steady and dynamic patterns. All statistics for this figure are calculated using a two-tailed two-sample Student's *t* test (*P < 0.05; **P < 0.01, ***P < 0.001). (**E**) FRET heatmap histograms generated from the dynamic traces. (**F**) Transition density plot (TDP) of the corresponding protein conditions.

Next, we asked if the discrete steps induced by POT1 and TRF2 persist in the presence of other shelterin components, TPP1 and TIN2, which physically interact with and bridge POT1 and TRF2. We performed sequential measurement by first adding POT1-TPP1 complex followed by TRF2 and TIN2 to the four repeat telomeric duplex with 4R overhang (Figure 6A). POT1–TPP1 complexes generated FRET fluctuations similar to the pattern seen in POT1 only (Figure 6B). Our previous study on POT1 and TPP1 reported a significantly lower range of FRET fluctuations (0.2–0.4) due to the different dye positions which are substantially less sensitive in capturing the wide range of motion that we probe here (25). The addition of TRF2 broadened the FRET peak without changing the peak position compared to the POT1-TPP1 bound state, as seen before (Figure 6B). Strikingly, the addition of TIN2 broadened and flattened the FRET histogram significantly, with values ranging from low to high FRET (Figure 6B). The single molecule traces for the POT1–TPP1 complex bound with 4R overhang showed sharp FRET transitions between 0.3 and 0.9, which is far more pronounced than our previous observation, but otherwise consistent (Figure 6C) (25). Further analysis revealed that the POT1–TPP1 complex also induces two patterns of dynamic FRET fluctuation whereas addition of TRF2 increased the overall dynamic molecules (Figure 6D). Furthermore, addition of TIN2 enhanced the dynamics to high FRET levels similar to the POT1–TPP1–TRF2 complex (Figure 6C, D). To directly compare the overall fluctuation dynamics of all shelterin protein conditions, we generated a FRET heatmap by combining single molecule traces which exhibit FRET fluctuations. The progression from POT1 alone to POT1–TPP1, POT1–TRF2, POT1–TPP1–TRF2, POT1–TPP1–TRF2–TIN2 is that the FRET peak becomes further spread out and more evenly distributed with FRET values between 0.2 and 0.9 FRET (Figure 6E). To test if a similar stepwise motion is present, we fitted our traces to a HaMMy algorithm to generate TDP for each successive case. The TDP demonstrates that the added proteins, TPP1 and TIN2 smooths out the steps into more finely divided states from the four sharply distinct steps that we observed for POT1–TRF2 (Figure 6F). Based on the same FRET range of motion we see in the presence of all four proteins, the emerging picture is that while the overall motion is still driven by POT1–TRF2 contacts, the accompanying proteins TPP1 and TIN2 act to make the motions less stepwise but more evenly spread.

### TRF2 attenuates POT1 association

**Figure 7.**
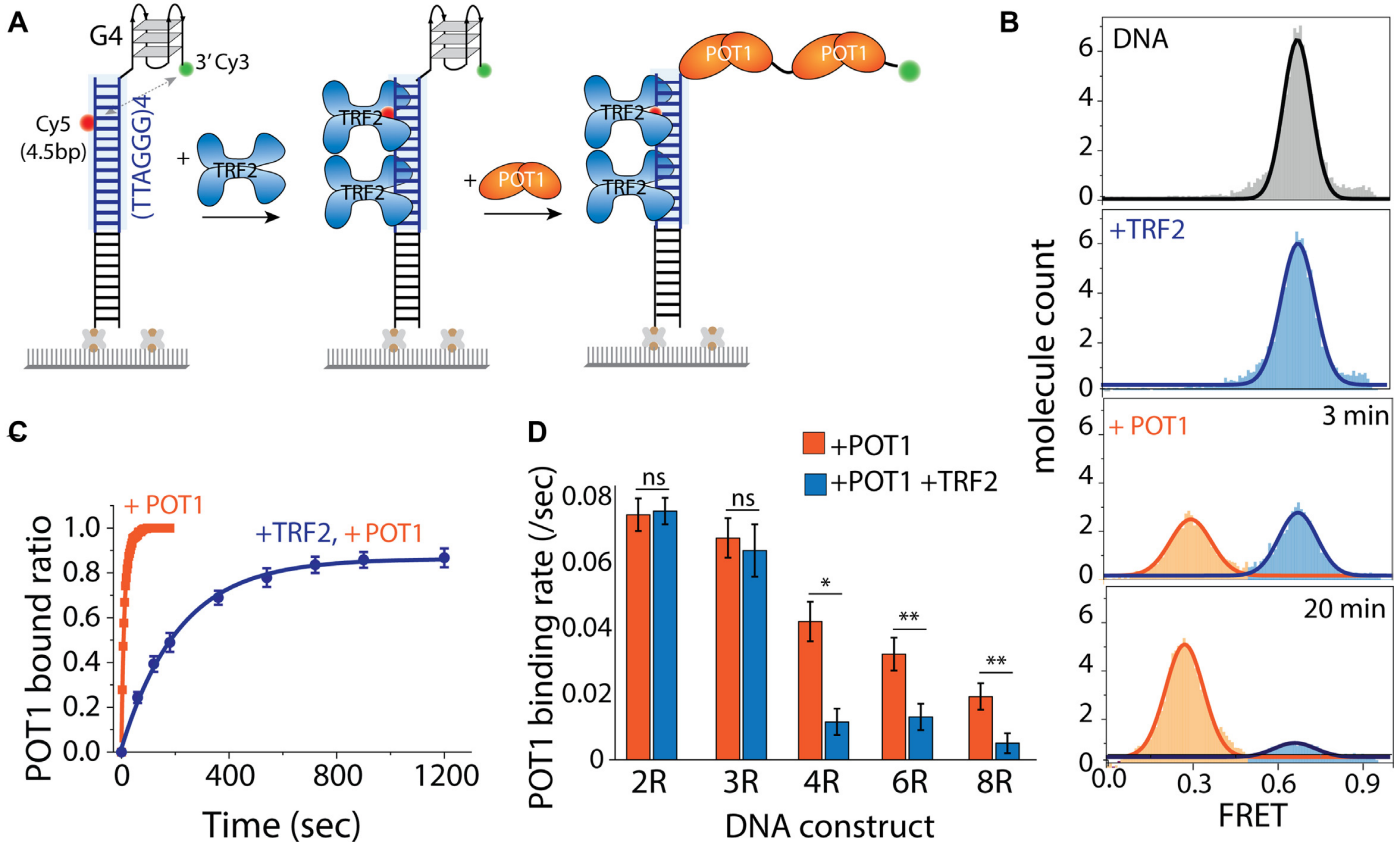
Telomeric duplex bound TRF2 delays POT1 binding to telomeric overhangs. (**A**) Schematic model of telomeric duplex (four repeats of TTAGGG tract) with the G4/4R overhang, sequentially binding TRF2 at the duplex followed by POT1 at the overhang. (**B**) The FRET histograms of telomeric duplex with 4R overhang (top), TRF2 bound condition (second from top), and POT1 binding at 3 and 20 min, respectively (bottom two). (**C**) Single-exponential fitting of POT1 bound fraction in the absence and presence of TRF2. (**D**) The bar graph represents the corresponding POT1 binding rate in the absence and presence of TRF2 of different overhang. All statistics for this figure are calculated using a two-tailed two-sample Student's *t* test (ns, not significant; *P < 0.05; **P < 0.01).

So far, we applied POT1 first followed by TRF2. We sought to reverse the order to depict a situation in which TRF2 engages with the telomeric duplex first then POT1 accesses the overhang (Figure 7A). TRF2 (25 nM) binding to telomeric duplexes did not change the FRET peak (at ∼0.65) as described previously (Figure 7B). When POT1 (50 nM) was added, the FRET peak displayed an extremely delayed shift to low FRET (∼0.3) indicating inefficient binding of POT1. Unlike the few seconds that POT1 takes to bind an overhang with unbound duplex (Figure 1), it took 3 minutes to observe ∼50% binding and 20 minutes to reach ∼90% bound fraction (Figure 7B). For kinetics analysis, the bound fractions (0.3 FRET peak) of POT1 alone and TRF2 followed by POT1 were collected over time and fitted to exponential decay to obtain the rate of binding (Figure 7C). The same experiment was performed with a non-telomeric duplex where no TRF2 is expected to bind, and revealed the same POT1 binding rate (Supplementary Figure S20). Hence, this clearly demonstrates that the presence of TRF2 at the duplex interferes with and delays the POT1 binding to the telomeric overhang.

Next, we asked if the delayed binding was due to TRF2 interacting with the G4 structure formed at the overhang. We reasoned that TRF2 as a key factor at the ds/ss junction of telomere may be contacting the overhang structure although it does not change the conformation of the overhang (Figure 7B). To test this effect, we applied the same sequence of TRF2 followed by POT1, to 2R and 3R, which don’t form higher order structures, and to 6R and 8R, which can form G4 structures (53). First, we compared the rate of POT1 binding and found that the rate decreases as a function of overhang length (Figure 7D, orange bars). In the presence of TRF2 bound at the duplex, the POT1 binding rate to 2R and 3R were comparable to POT1 only (Figure 7D, blue bars). By contrast, TRF2 presence with POT1 displayed a marked decrease in 4R, 6R, and 8R, suggesting that the TRF2-duplex interacts with the structured telomeric overhang and thereby, attenuates the POT1 binding (Figure 7D, blue bars). Interestingly, when POT1 and TRF2 were added simultaneously to all constructs (2R to 8R), we found a similar delayed POT1 binding for 4R, 6R and 8R (Figure 7D), consistent with the higher binding rate of TRF2-duplex (∼4-fold) than POT1-overhang (Supplementary Figure S14).

## DISCUSSION

**Figure 8.**
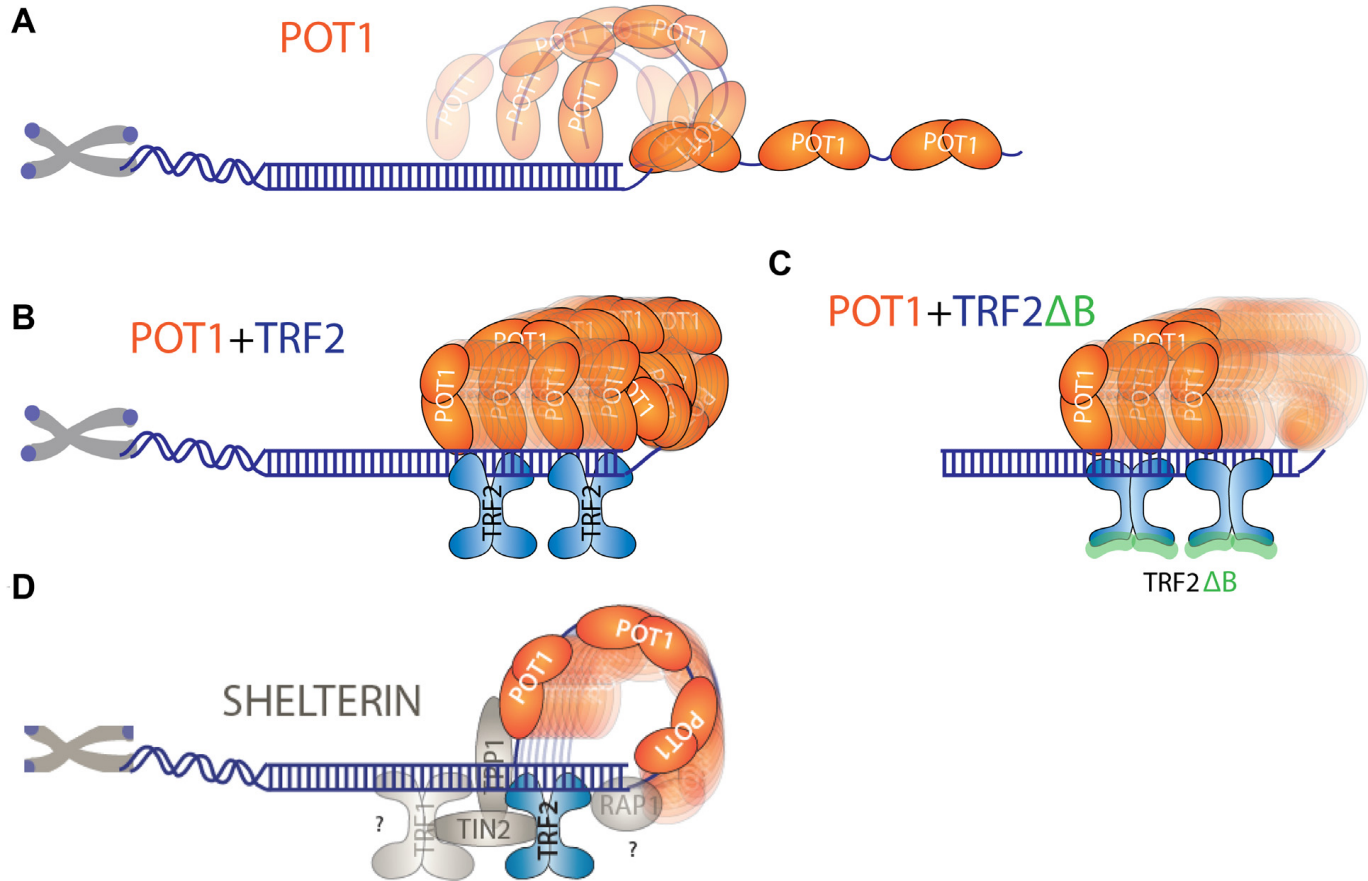
Proposed dynamic model of POT1 and POT1 with TRF2 bound to telomeric DNA. (**A**) POT1 bound telomeric overhang showing dynamics. (**B**) TRF2 recruit POT1 bound 3′ end towards the junction and moving up and down along the duplex axis. (**C**) TRF2ΔB doesn’t recruit POT1 bound 3′ end towards the junction. (**D**) Shelterin components bridging interactions within the proteins bound at telomeric DNA.

In this work, we report an unanticipated dynamic nature of the POT1-overhang complex which makes frequent contact with the duplex, suggesting an inherent bendability or flexibility of the telomeric structure. Remarkably, such conformational dynamics of the POT1-overhang is dramatically increased by TRF2 bound at the telomeric duplex *in cis*. Intriguingly, the movement is systematically coordinated by the two proteins, evidenced by the discrete stepwise FRET changes we monitored only in the presence of both POT1 and TRF2. The number of steps correspond to the number of TRF2 homodimers bound to the duplex and the same steps persist regardless of the length of the POT1-bound overhang. Based on this result, we propose a dynamic looping mechanism by which TRF2 actively recruits the tip of the POT1-overhang and enables a stepwise movement up and down the axis of telomeric duplex (Figure 8).

Telomere end-binding protein POT1 disrupts G4 in a stepwise manner, one OB-fold at a time, and thereby results in a highly stable complex (Figure 1) (24,25). Unlike other ssDNA binding proteins, such as SSB, RecA and Rad51, POT1 binds telomeric overhang with an exquisite sequence specificity and produces a sharp kink (24,25,47,56,57). Despite the highly stable binding, POT1-induced telomeric overhang dynamics even at monomer bound states (Figure 2), was not as obvious and was underappreciated in our previous study, in part due to different dye labelling positions that induced less pronounced FRET changes (25). The POT1-induced telomeric overhang dynamics are uniquely distinguished from other classes of ssDNA binding proteins including RPA which diffuses along ssDNA (58), as well as RecA and Rad51 which forms a stable helical filament and undergoes ATP-dependent dynamic assembly and disassembly (47,56,57). The pattern of FRET fluctuations induced by POT1 bound to G4 overhangs suggests transient and dynamic loop formation (Figure 2). In light of the molecular bond rotations that exhibit a faster time scale than the dynamic conformational changes we detect here, the transition to high FRET seen in Dynamic-II represents two monomer POT1 imparting dynamic conformational change in the telomeric overhang, likely using a flexible two guanine spacer in between the two POT1 units as a pivot point (Figure 2). POT1 binding to overhangs is linked to telomeric vs. non-telomeric duplexes which yielded the same result, indicating that the transient looping activity is an intrinsic property of the POT1-overhang complex, independent of the duplex sequence. The dynamic property of the POT1 bound telomere overhang may play a role in diminishing the overhang access to telomerase (59).

Our results revealed an intriguing property of TRF2 in recruiting the POT1-overhang and inducing a stepwise movement up and down the telomeric duplex axis without requiring an external source of energy (Figure 3). This is reminiscent of the t-loop/D-loop that forms in the context of telomeric duplexes where the overhang invades by assistance of shelterin components (9,32). Nevertheless, it was shown previously that TRF2 and RTEL1, but not POT1 are the two key factors required for T-loop formation (55,60). The emerging picture based on the FRET fluctuation pattern is that the tip of the POT1-overhang complex makes physical contact with each homodimer of TRF2 bound on the duplex, rendering four steps corresponding to four TRF2 or two steps for two TRF2 units. This motion is transient and dynamic, yet persistent, giving rise to dynamic looping where the looped circle undergoes tightening and expanding continuously. Such movement requires that both TRF2 and POT1 proteins interact with the telomeric duplex and overhang, respectively as TRF2 applied *in-trans* to non-telomeric duplex cannot recapitulate the dynamic looping activity. One plausible scenario is that such dynamic motion is used for assembling shelterin in which POT1 and TRF2 need to be linked to adjacent proteins including TPP1, RAP1, TRF1 and TIN2 (9,10). Consistently, previous reports suggested that TRF2 uses one-dimensional sliding to find partner proteins and assemble into the shelterin complex (13,34). Further, POT1 and TRF2 may interact with each other to form a complex with telomeric DNA to maintain the telomeric length, (35) and POT1 are TRF2 are co-localized within a larger complex at the telomere (12,61). Our study adds to the previous findings by demonstrating that TRF2 and POT1, upon binding the telomeric duplex and overhang, respectively, generate a highly dynamic motion with discrete steps which may lead to finding a correct configuration assisted by other shelterin components (Figure 8). Our observations with the TRF2ΔB mutant is in agreement with the previous finding that TRF2, but not TRF2ΔB binds branched DNA to protect three- and four-way junction (55,62). In addition, our result suggests a role of TRF2 basic domain in recruiting the POT1 bound 3′ end to the telomeric junction thereby forming a loop conformation (Figure 8).

There are four possible contributing aspects towards the driving force of the stepwise movement. First is the least likely scenario in which the complementarity between the overhang and the C-rich strand in duplex can generate base pairing, but this is not expected based on the way POT1 binds the Watson-Crick edges of the bases. Second, POT1 can induce dynamic bending of overhangs as shown, yet POT1 alone produced dynamic patterns (Dynamic-I, -II) which differed from the four steps observed in the presence of TRF2. Third, TRF2 can recruit the overhang strand to stimulate invasion, but TRF2 without POT1 did not produce any FRET changes, which led us to use PIFE to probe its association (Supplementary Figure S14). Fourth, it is also plausible that a protein-protein interaction between the C-terminal domain of POT1 and TRF2 is the driving force of the observed steps. This interpretation is consistent with both two and four TRF2 binding sites which generate two and four steps, respectively. It is clear from our observations that both POT1 and TRF2 positioned in the overhang and duplex of telomere, respectively, are required for this looping activity. The base pairing may play a role, but only in the context of both proteins engaged with the DNA. We note that amongst the five FRET states that constitutes the four steps, the lowest FRET value corresponds to the POT1 bound extended overhang without engaging with TRF2. Therefore, the dynamic motion includes excursions to the un-looped state interspersed with the differently looped states.

One of the most surprising conformations was revealed by the extremely high FRET (∼0.9) state observed in DNA with a long overhang such as 6R and 8R (i.e 36–48 nucleotides). POT1 alone induced mostly low FRET states which agrees with the tandemly bound POT1 along the overhang. However, in the presence of both POT1 and TRF2, the FRET level rose to 0.9, clearly indicating a loop which forms by bringing the 3′ end to ss/ds junction (Figure 4). This resembles the telomere capping model in which the 3′ end of the telomere loops to invade the duplex (32). In addition, the shelterin complex is positioned near the 3′ end where TPP1 and POT1 regulates telomerase recruitment to the 3′ end (63,64). POT1 has a higher affinity to the 3′ end sequence TTAG, to stabilize the shelterin complex (65). Hence the 3′ end is the key moiety of telomeric overhang which plays a critical role in shelterin maintenance and telomerase recruitment.

Many studies provided evidence that six shelterin proteins associate with each other to coat the telomeric DNA and safeguard the chromosome ends (1,9). While POT1 and TRF2 are the resident proteins on the telomere overhang and duplex, respectively, TIN2 and TPP1 are connector proteins situated in between to link POT1 and TRF2 (10). In agreement, while POT1 and TRF2 bind the telomeric substrate independently, dictated by the sequence specific interaction, TIN2 and TPP1 only associate with the telomeric complex via engaging with POT1 and TRF2. Therefore, the addition of TIN2 and TPP1 to POT1 and TRF2 should reflect the role of the liaison proteins that may modulate the mechanistic link between the resident proteins, POT1 and TRF2. In this light, our observation of smaller and more finely subdivided FRET steps induced by TIN2 and TPP1 can be interpreted as the role of these connecting proteins in fine-tuning the otherwise abrupt stepwise motion generated by POT1 and TRF2. Additionally, such fine-tuned movement can also be further modulated by additional shelterin components, which warrants future study. We note that the highly dynamic FRET fluctuations that we observe in all scenarios including POT1-overhang, POT1–TRF2, POT1–TRF2–TIN2–TPP1 are not due to transient binding and unbinding of the protein constituent as all assays were done with thorough buffer wash of unbound proteins. Therefore, the dynamics we observe likely arise from the inherently dynamic nature of the shelterin bound telomeric DNA with agreement from the previous finding (9,13,34). The broad range of FRET fluctuation (0.2–0.9) induced by the four proteins reflect that the joint molecules TIN2 and TPP1 do not constrain POT1–TRF2 coordinated movement. Rather, they act as flexible linkers that enable dynamic motion of POT1 bound overhang with respect to the TRF2 bound telomere duplex.

Interestingly, we found that POT1 binding was significantly delayed in the presence of TRF2 in the duplex. The delay was only observed at 4R, 6R and 8R but not at 2R and 3R (Figure 7). TRF2 preferentially binds the ds/ss junction of telomeres, near the POT1 binding site (10,31). Therefore, TRF2 may have a natural binding affinity to G4 or G4 containing higher ordered structures to attenuate POT1 binding. Indeed, our kinetic analysis revealed that the binding rate of the TRF2-duplex is ∼4 times faster than the POT1-overhang, which makes TRF2 associate prior to POT1 binding (Supplementary Figure S14). Further, the cellular POT1 concentration is about ∼5–10-fold lower than TRF2, and the *K*_D_ of POT1 is higher than TRF2 (9,13,24), raising the possibility of this scenario. Indeed, POT1 is recruited to the telomere via TRF1/TRF2, TIN2 and TPP1 in cells. Therefore, the ssDNA binding activity of POT1 is not sufficient for telomeric localization independently. Importantly, the complex formation could potentially limit the conformational flexibility of overhang associated POT1 in cells. Therefore, the fast and flexible movement that we observe in this study may represent an accentuated scenario of an enhanced movement which still reveals the inherent interactions coordinated by the shelterin proteins.

One important function of shelterin is to protect telomeres from unwanted degradation and end-to-end fusion (1,2). Shelterin complexes harbouring both TRF2 and POT1 plays a pivotal role in end protection by preventing activation of the ATM and ATR kinase DNA damage response (14,55). Evidence suggests that POT1 and TRF2 search for telomeric ss/ds junctions by three-dimensional diffusion and upon binding, POT1 scans along the ss-overhang to stabilize the 3′ end (Figure 8D) (10,13,34). This mobile property generated by POT1 and TRF2 that we report here may contribute to the mechanism of shelterin assembly, telomere protection, and telomerase recruitment.

## Supplementary Material

gkab1123_Supplemental_File
