# Supplementary material for: TRF2 promotes dynamic and stepwise looping of POT1 bound telomeric overhang
Source: Nucleic Acids Res. 2021 Nov 25;49(21):12377–93. doi: 10.1093/nar/gkab1123 (PMC8643667; doi:10.1093/nar/gkab1123)
Supplement: gkab1123_Supplemental_File [file gkab1123_supplemental_file.pdf]

SUPPLEMENTARY INFORMATION

TRF2 promotes dynamic and stepwise looping of POT1 bound telomeric overhang

Tapas Paul[1], Wilson Liou[1], Xinyi Cai[1], Patricia L. Opresko[2] and Sua Myong[1,3,*]

Author Affiliations:
[1]Department of Biophysics, Johns Hopkins University, Baltimore, MD 21218, USA.
[2]Department of Environmental and Occupational Health, University of Pittsburgh.
[3]Physics Frontier Center (Center for Physics of Living Cells), University of Illinois, 1110 W. Green St., Urbana, IL, 61801, USA.
*To whom correspondence should be addressed: Email: smyong@jhu.edu; Tel: 410-516-5122; Fax: 410-516-4118.

Supplementary information includes:
Supplementary Table 1
Supplementary Figures, S1-S20

Supplementary Table 1. DNA Oligonucleotides (5' to 3') used in this experiment.

2R/3'Cy3/	TGG CGA CGG CAG CGA GGC (TTA GGG)$_2$ /3'Cy3/
3R/3'Cy3/	TGG CGA CGG CAG CGA GGC (TTA GGG)$_3$ /3'Cy3/
3R TTAG/3'Cy3/	TGG CGA CGG CAG CGA GGC (TTA GGG)$_3$ TTAG/3'Cy3/
4R/3'Cy3/	TGG CGA CGG CAG CGA GGC (TTA GGG)$_4$ /3'Cy3/
6R/3'Cy3/	TGG CGA CGG CAG CGA GGC (TTA GGG)$_6$ /3'Cy3/
8R/3'Cy3/	TGG CGA CGG CAG CGA GGC (TTA GGG)$_8$ /3'Cy3/
10R/3'Cy3/	TGG CGA CGG CAG CGA GGC (TTA GGG)$_{10}$ /3'Cy3/
12R/3'Cy3/	TGG CGA CGG CAG CGA GGC (TTA GGG)$_{12}$ /3'Cy3/
4R/int-Cy3/	TGG CGA CGG CAG CGA GGC (TTA GGG)$_2$/int-Cy3/(TTA GGG)$_2$
R8	TGG CGA CGG CAG CGA GGC (TTA GGG)$_8$ /3'
4R_GtoC/3'Cy3/	TGG CGA CGG CAG CGA GGC (TTA GGG)$_3$ TTACGG /3'Cy3/
4R_GtoA/3'Cy3/	TGG CGA CGG CAG CGA GGC (TTA GGG)$_3$ TTAAGG /3'Cy3/
4R_GtoT/3'Cy3/	TGG CGA CGG CAG CGA GGC (TTA GGG)$_3$ TTATGG /3'Cy3/
T6_2R_T6/3'Cy3/	TGG CGA CGG CAG CGA GGC TTT TTT (TTA GGG)$_2$ TTT TTT /3'Cy3/
Top0/5'Cy5/18merBio/	/5'Cy5/GCC TCG CTG CCG TCG CCA/3'Bio/
Top4.5/5'Cy5/18merBio/	GCC T/Cy5/CG CTG CCG TCG CCA/3'Bio/
Top4.5/5'Cy5/C2-18merBio/	CCC T/Cy5/AA CCC TAA GCC TCG CTG CCG TCG CCA/3'Bio/
Top4.5/5'Cy5/C4-18merBio/	CCC T/Cy5/AA (CCC TAA)$_3$ GCC TCG CTG CCG TCG CCA/3'Bio/
C4-/18merBio/	(CCC TAA)$_4$ GCC TCG CTG CCG TCG CCA/3'Bio/
Top7.5/5'Cy5/18merBio/	GCC TCG C/Cy5/TG CCG TCG CCA/3'Bio/

SUPPLEMENTARY FIGURE 1

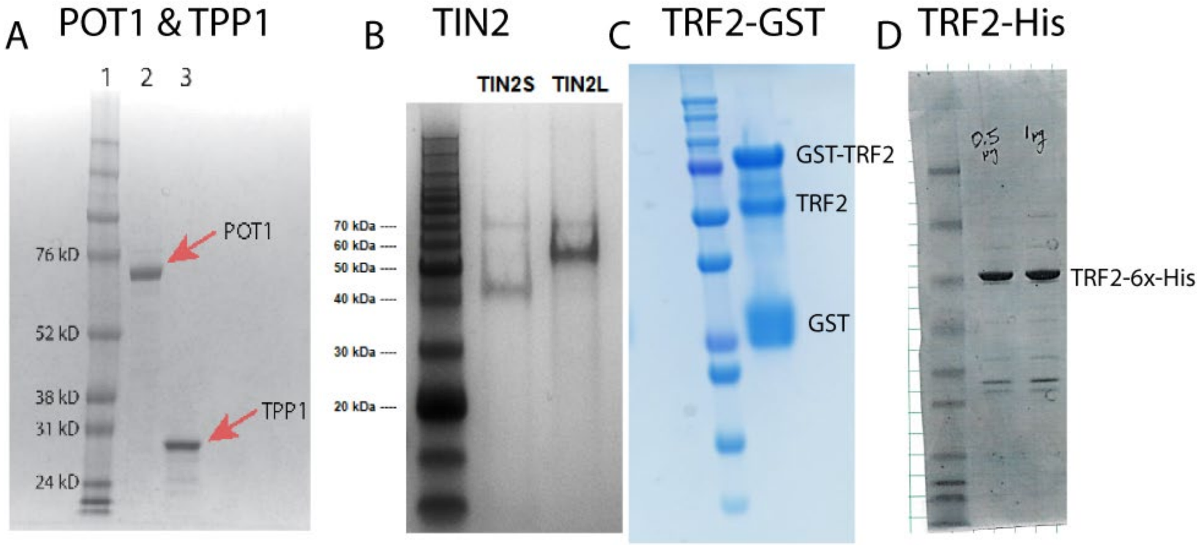

Figure S1. After affinity and size exclusion chromatography purification, POT1 and TPP1 proteins were analyzed on an SDS-PAGE gel with coomassie staining for purity. **(A)** Lane 1 contains the full range rainbow molecular marker (Cytiva), with 2.5 µg of purified POT1 protein in lane 2, and an equal amount of purified TPP1 protein in lane 3. **(B)** The TIN2 gel form GenScript, where TIN2S and TIN2L are the short and long version of TIN2. We used TIN2L protein throughout the experiment. **(C)** The SDS-PAGE gel of purified TRF2-GST in which GST was partially digested. **(D)** The TRF2-hexa histidine purification gel. We show that TRF2-GST and TRF2-histag produced the same experimental outcome.

SUPPLEMENTARY FIGURE 2

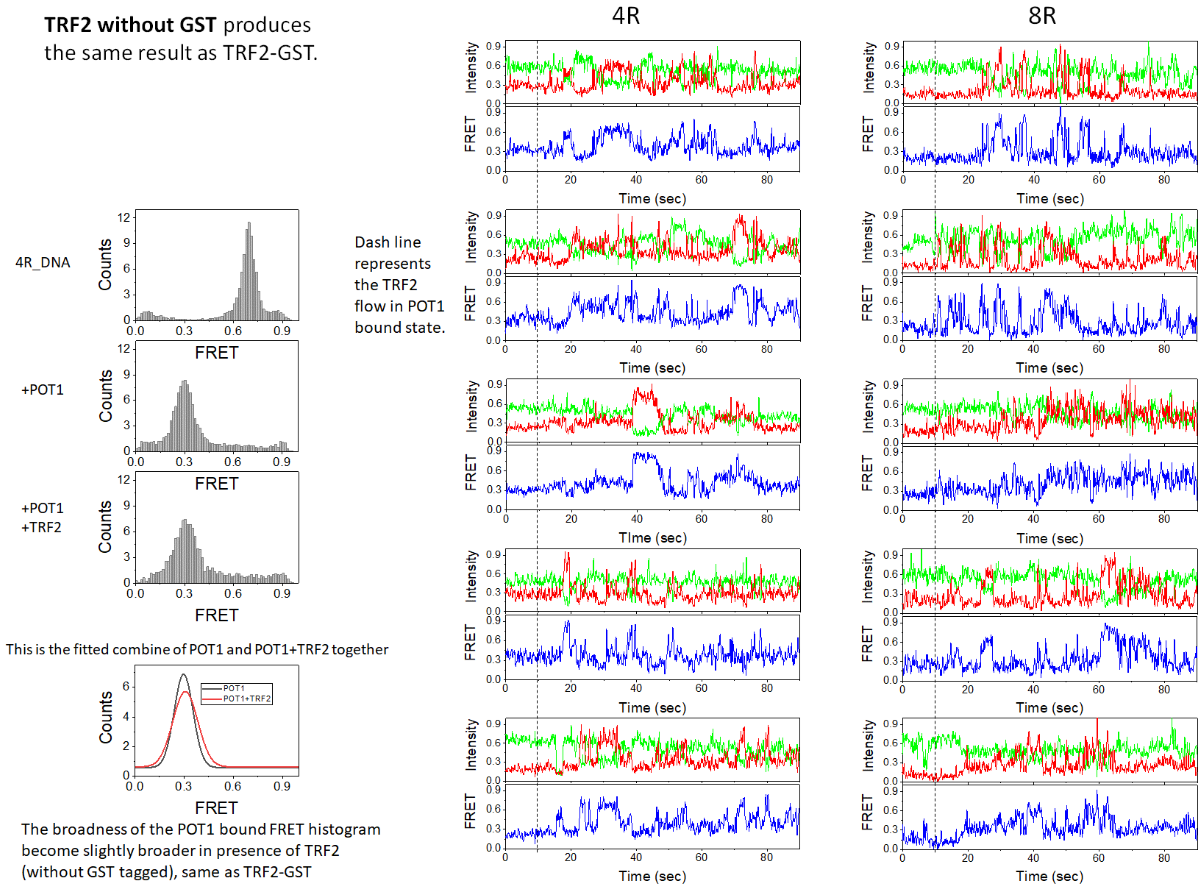

Figure S2. TRF2-histag without the GST tag produces the same effect on increasing POT1-overhang dynamics. The FRET experiment in which POT1 and TRF2 are added to 4R and 8R overhang display the same FRET pattern i.e broadened FRET histogram and substantially increased FRET fluctuation in the presence of TRF2. Therefore, we conclude that the TRF2-GST presented in main figures do not arise from GST tag on TRF2.

SUPPLEMENTARY FIGURE 3

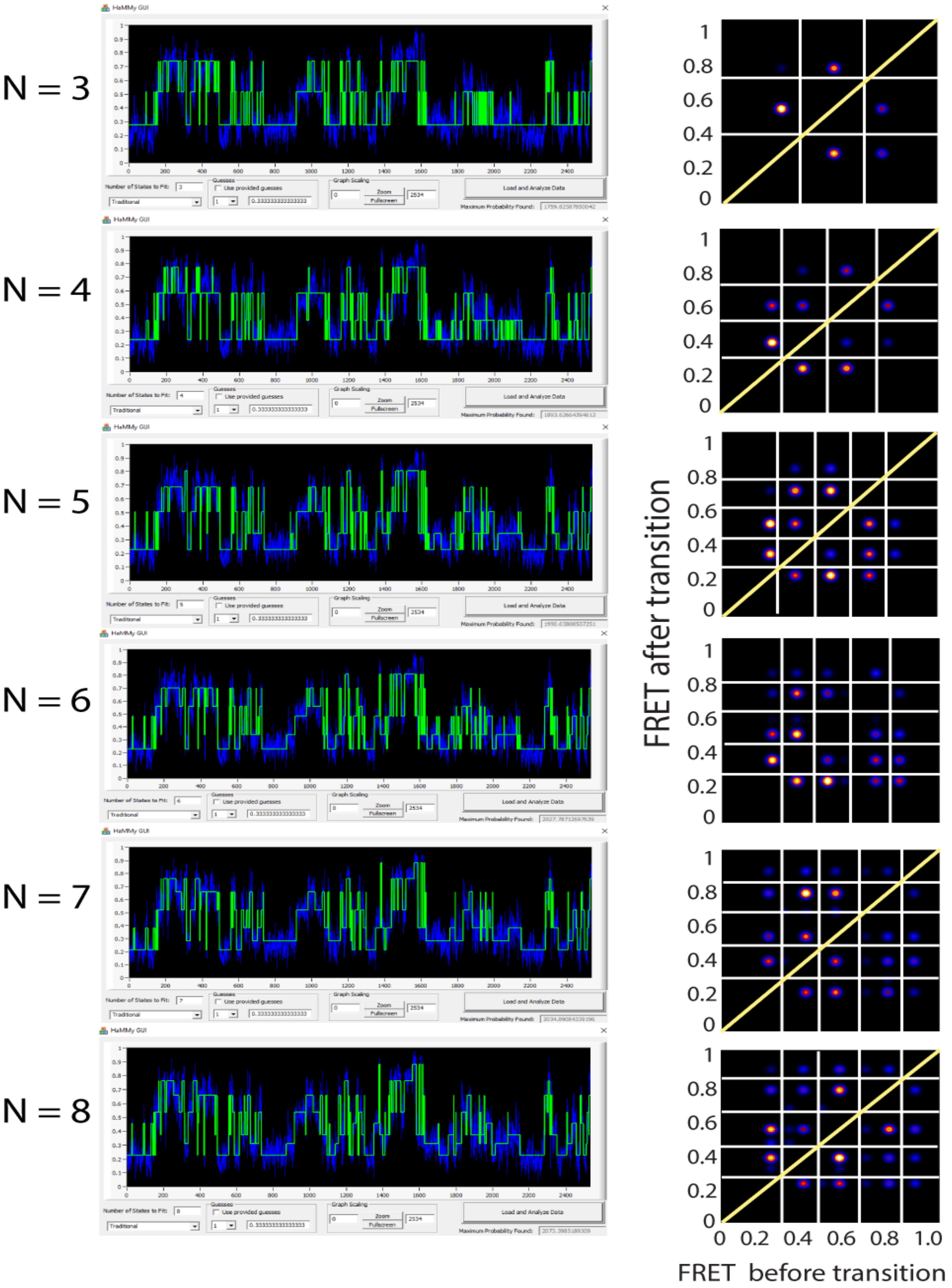

Figure S3. Exploring the HaMMy fitting and TDP generation of POT1 and TRF2 bound to 4R overhang with four repeats of TTAGGG containing duplex. Different number of state (N=3 to N=8) were applied for HaMMy fitting (left side) and the corresponding TDP were generated accordingly (right side). When the number is less than 5 (5<N), the different FRET state increases according to the number of state and when the number is 6 or greater than 6 (N≥6), the different states converge to the same five FRET states (four steps in TDP). Detailed description of HaMMy fitting and TDP generation are included in method.

SUPPLEMENTARY FIGURE 4

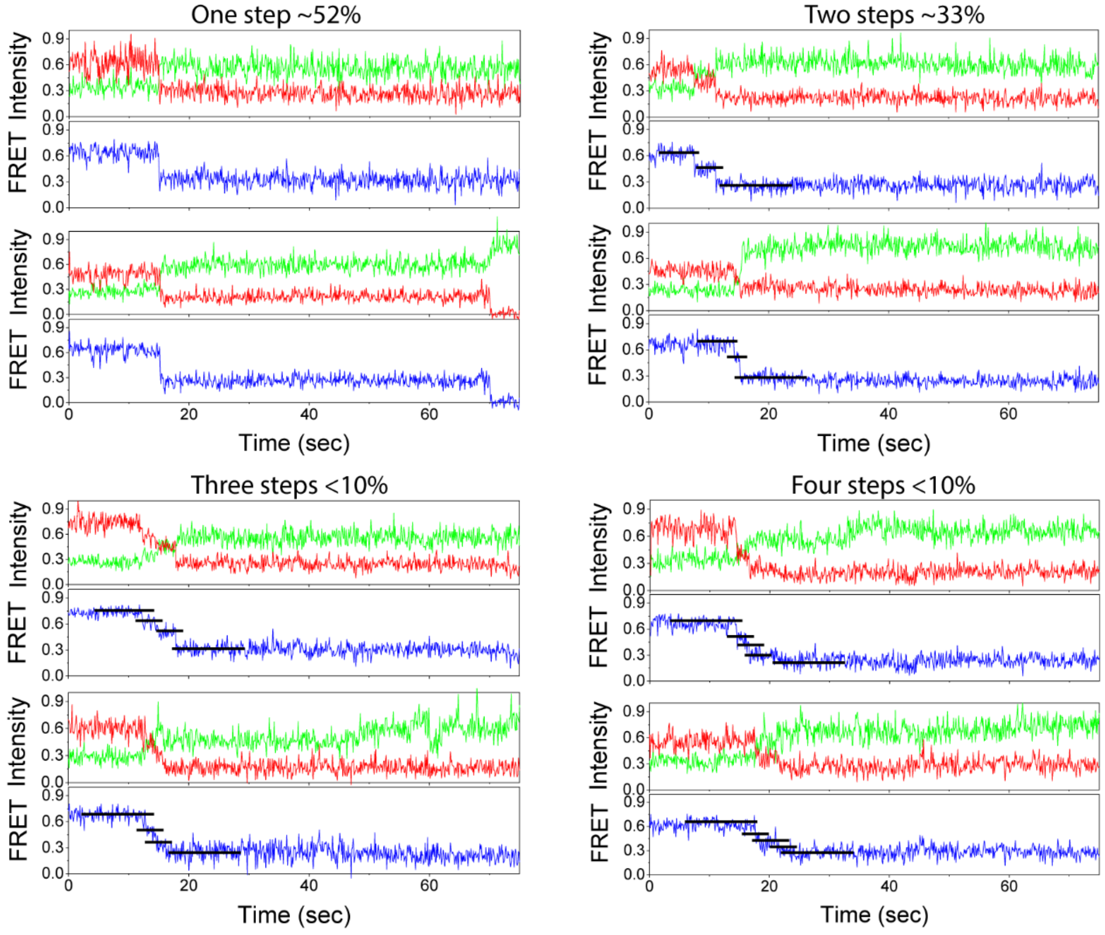

Figure S4. Single-molecule FRET traces of real-time POT1 binding to telomeric G4/4R overhang show one, two, three and four steps of FRET decrease respectively. POT1 flow at ~10 sec. The stepwise decrease of FRET state indicates the steps of POT1 binding that induce G4 unwinding.

SUPPLEMENTARY FIGURE 5

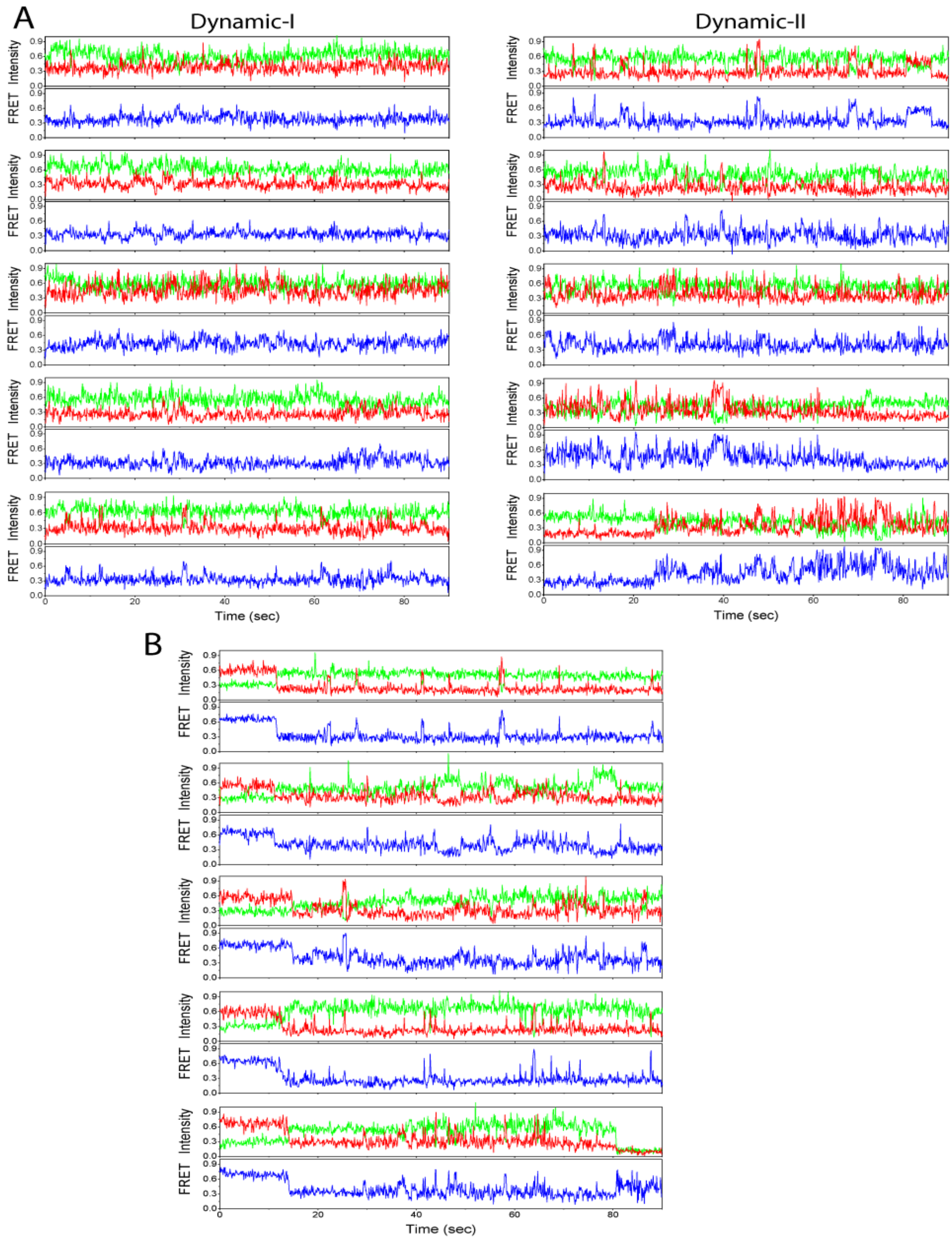

Figure S5. **(A)** Single-molecule dynamic FRET traces of POT1 bound telomeric G4/4R overhang are categorized as Dynamic-I (FRET ~0.2-0.6), and Dynamic-II (FRET ~0.2-0.9) based on the FRET fluctuation pattern. Dynamic-I displays low FRET to mid FRET transition whereas Dynamic-II pattern exhibits sharp low to high FRET peaks. **(B)** Representative single molecule real-time traces of POT1 binding seen as rapid FRET decrease between 10 and 20 seconds i.e. POT1 flow at ~10 sec and FRET jump from FRET ~0.7 to FRET ~0.3 indicates POT1 binding. This is subsequently followed by G4/4R overhang dynamics.

SUPPLEMENTARY FIGURE 6

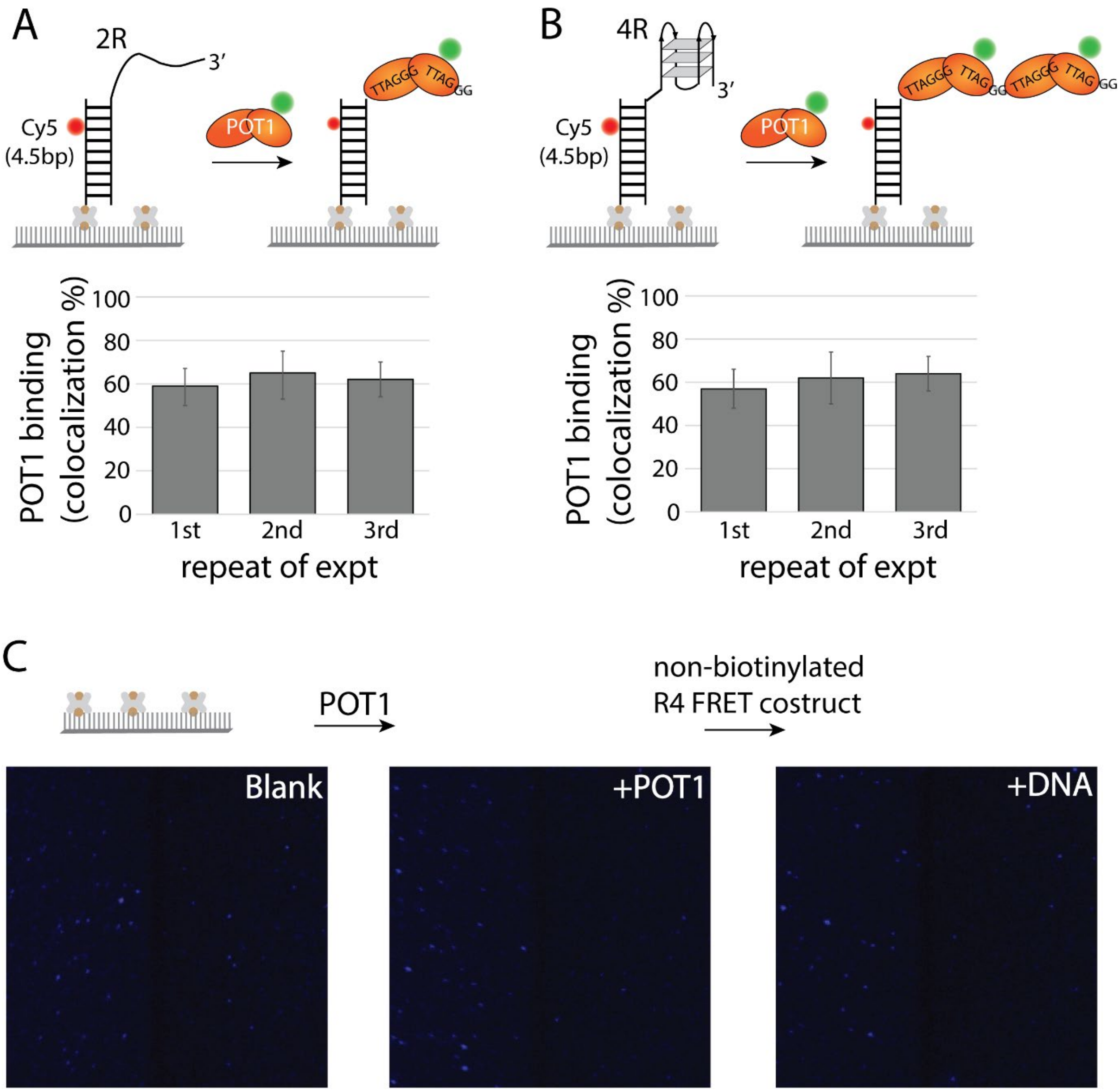

Figure S6. POT1 does not bind to the PEG coated slide surface non-specifically. **(A & B)** Schematic model of Cy3-labeled POT1 binding to Cy5 labeled 2R **(A)** or 4R **(B)** overhang respectively. POT1 colocalization with 2R and 4R was probed by overlapping signals of Cy3 and Cy5 molecules performed in three separate trials. The colocalization of Cy3 and Cy5 signal ~60-65% indicates that POT1 doesn't bind nonspecifically to the slide surface as the POT1 labelling efficiency was ~72% (described in method section). **(C)** To further test if there any nonspecific binding of POT1, POT1 (50 nM) was added to the biotin-NeutrAvidin coated PEG surface followed by non-biotinylated 4R FRET construct (1 nM). If POT1 is non-specifically bound to surface, FRET signal will appear after addition of non-biotinylated 4R FRET construct. The representative field view of blank (left side), followed by POT1 addition (middle) and then non-biotinylated 4R FRET construct (right side) doesn't show any FRET signal which indicates no nonspecific binding of POT1 to surface.

SUPPLEMENTARY FIGURE 7

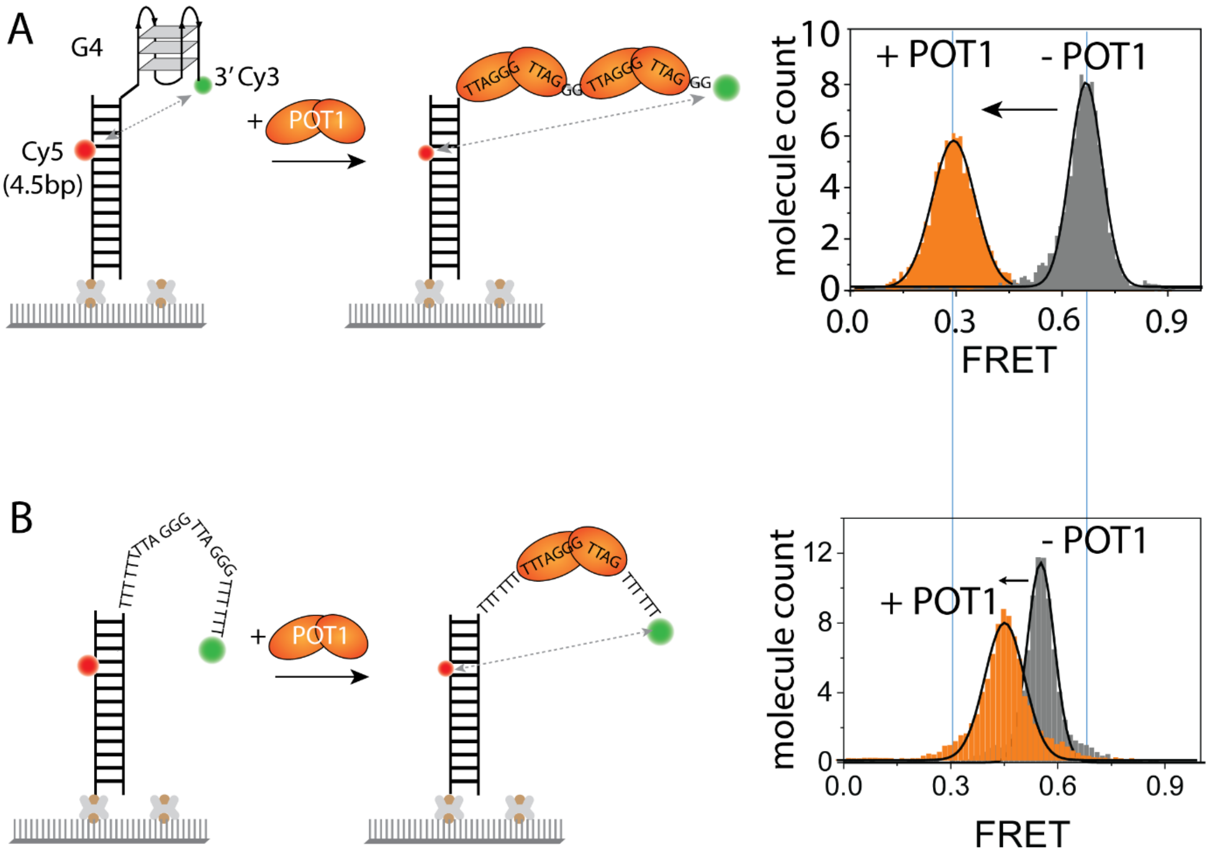

Figure S7. (A) The smFRET cartoon model of G4 where two POT1 can bind side by side. Beside contain the FRET histogram of G4 DNA before (~0.7) and after (~0.3) POT1 binding. **(B)** The smFRET cartoon model of T_6(TTAGGG)$_2T_6$ (24nt) where one POT1 can bind at the center. Beside contain the FRET histogram of DNA before (~0.55) and after (~0.45) POT1 binding.

SUPPLEMENTARY FIGURE 8

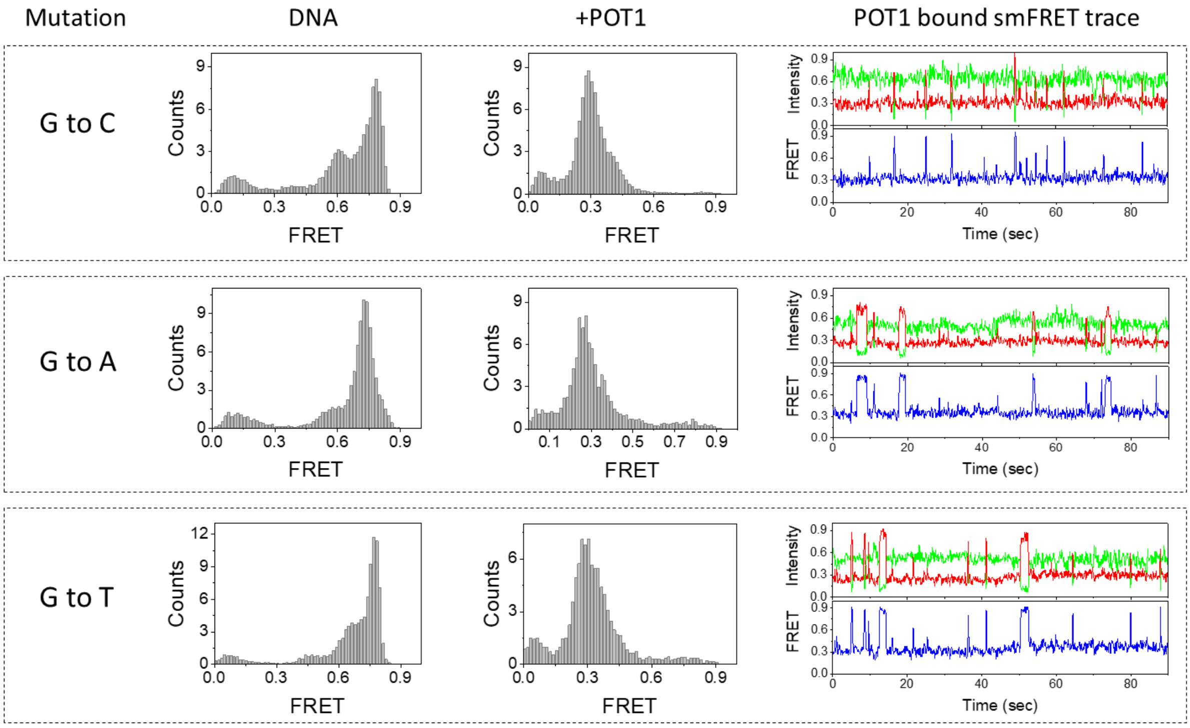

Figure S8. POT1 bound to mutated G4/4R show dynamic. The single nucleotide substitution is in 4R at G1 position of fourth repeat of TTAGGG, [(TTAGGG)$_3$ TTA**G**^{1}GG] of Top4.5 construct. The FRET histograms of DNA on the left column and POT1 bound histograms in the middle column are side by side for comparison along with representative smFRET traces of G to C, G to A and G to T mutations in G4 at G1 position of fourth repeat of TTAGGG.

SUPPLEMENTARY FIGURE 9

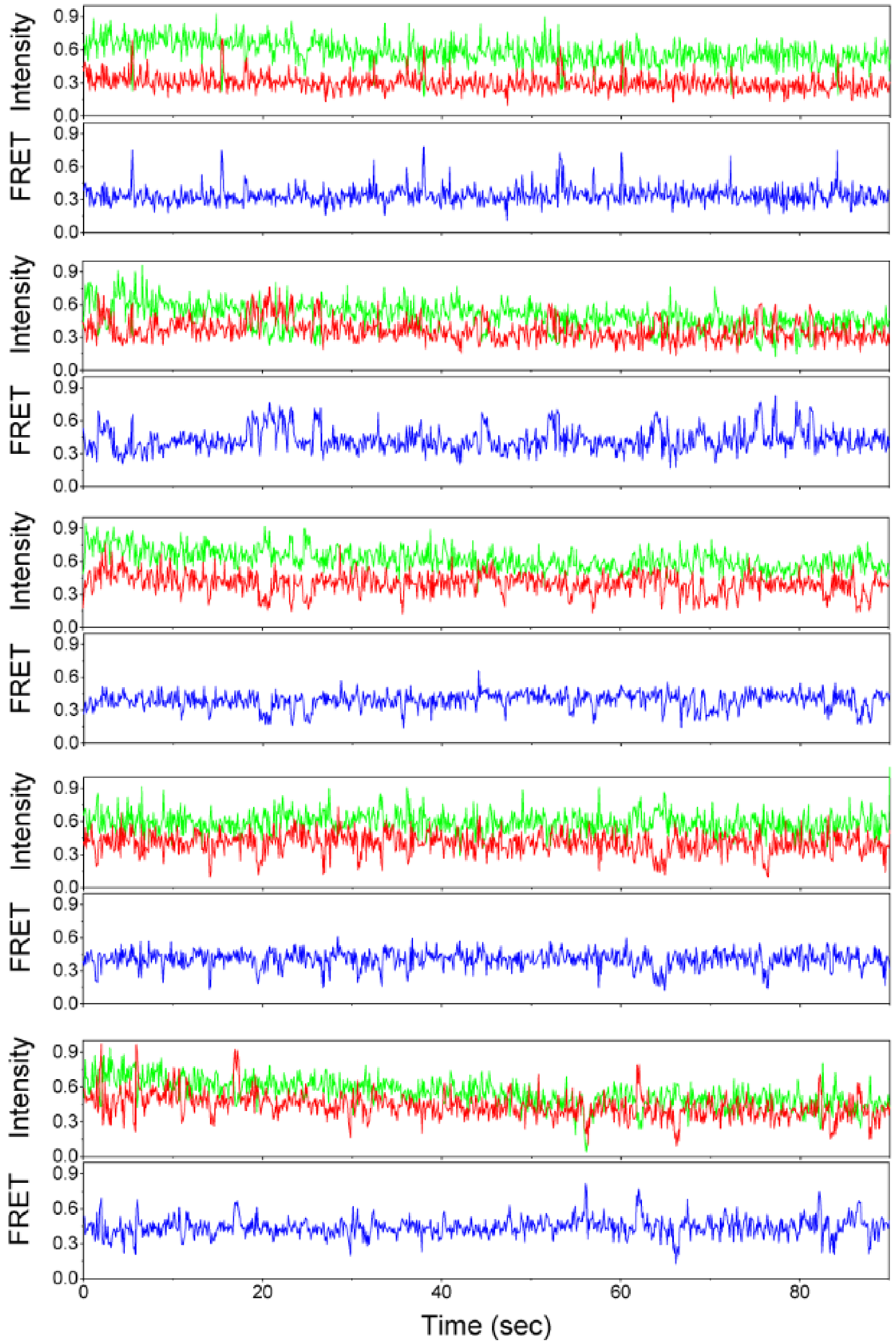

Figure S9. Representative single-molecule FRET traces of POT1 bound to Top0 construct show dynamics. This is the dye arrangement used in our previous study (Hwang et al, Structure 2012) in which FRET fluctuation dynamics is far less prominent than the Top4.5 construct used in this study. In fact, the dynamic fluctuation was not reported in our previous work. Therefore we conclude that the dye position is critical in detecting different range of motion in 3D wingspan of the molecule.

SUPPLEMENTARY FIGURE 10

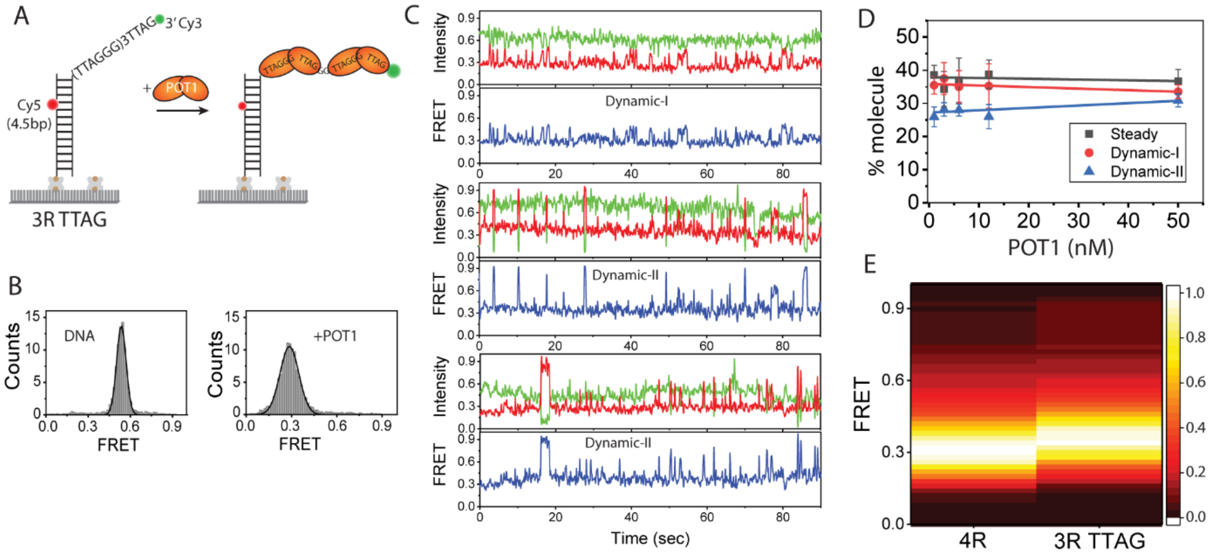

Figure S10. POT1 bound telomere (TTAGGG)3TTAG overhang shows the same dynamics as in 4R. **(A)** Schematic of smFRET experiment for POT1 binding to telomere 3R TTAG overhang (Top4.5 construct). **(B)** The smFRET histograms of 3R TTAG before and after POT1 binding. **(C)** Representative smFRET traces of POT1 bound to telomeric 3R TTAG (after wash of free protein) overhang showing Dynamic-I and Dynamic-II behavior. **(D)** Quantification of molecular behavior of POT1 bound 3R TTAG (steady vs. two types of dynamic) at the protein concentrations ranging from 1 to 50 nM. **(E)** FRET heatmap histogram of 4R and 3R TTAG constructs generated from the dynamic traces (keeping the bin size 0.2).

SUPPLEMENTARY FIGURE 11

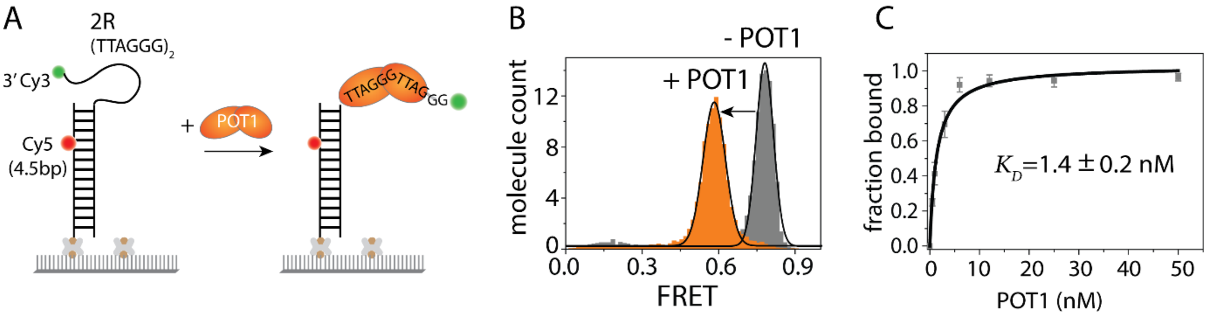

Figure S11. (A) Schematic smFRET model cartoon of before and after monomer POT1 binding to the telomeric 2R overhang where the acceptor dye (red) is labeled at top4.5 position. **(B)** The FRET histograms of 2R before and after POT1 binding. **(C)** Determination of the dissociation constant (K_D) of POT1 to 2R. We note that due to the extremely stable binding of POT1 to telomeric DNA, we cannot detect protein dissociation in our measurement. Therefore, the K_D plotted in (**C**) can be best described as the apparent dissociation constant.

SUPPLEMENTARY FIGURE 12

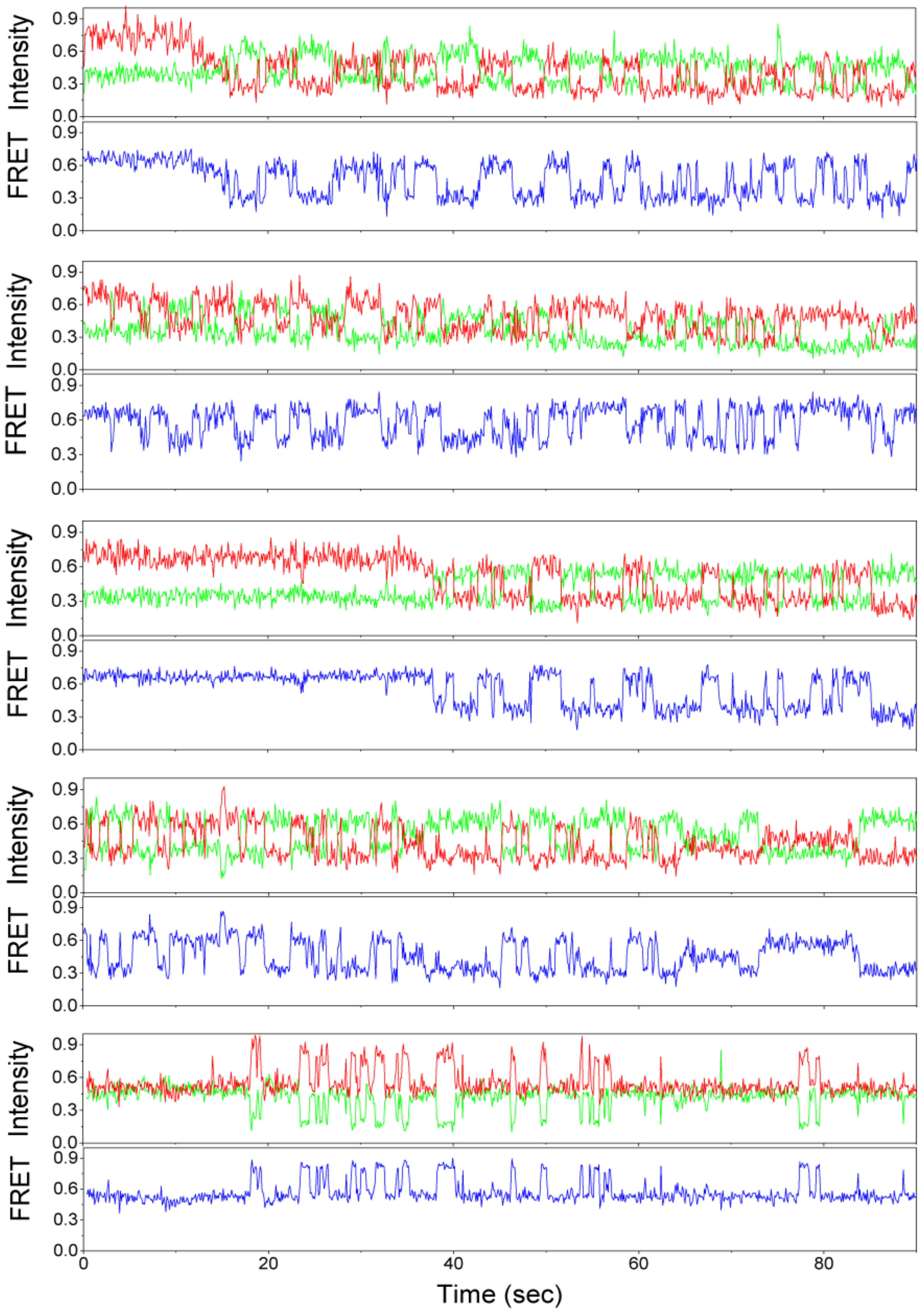

Figure S12. Single-molecule dynamic FRET traces of monomer POT1 bound to telomeric G2/2R overhang. This result clearly demonstrates that even a monomer POT1 displays dynamic conformational change when bound to a short 12 nucleotide ssDNA composed of two repeats of TTAGGG.

SUPPLEMENTARY FIGURE 13

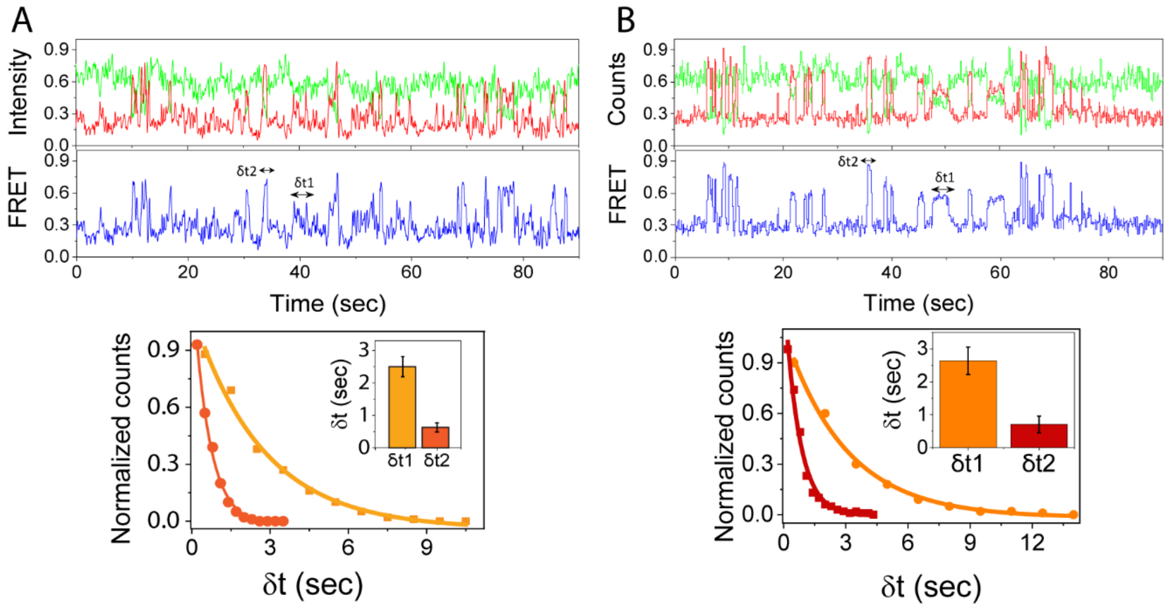

Figure S13. (A & B) The representative smFRET traces of POT1 bound to telomeric G4 overhang with non-telomeric duplex **(A)** and four TTAGGG repeat containing telomeric duplex **(B)**. Both traces show Dynamic-I (δt1) and Dynamic-II (δt2) and below contains the dwell time of the respective dynamic state. Here, the Dynamic-I and II are categorized by the FRET pattern as done before.

SUPPLEMENTARY FIGURE 14

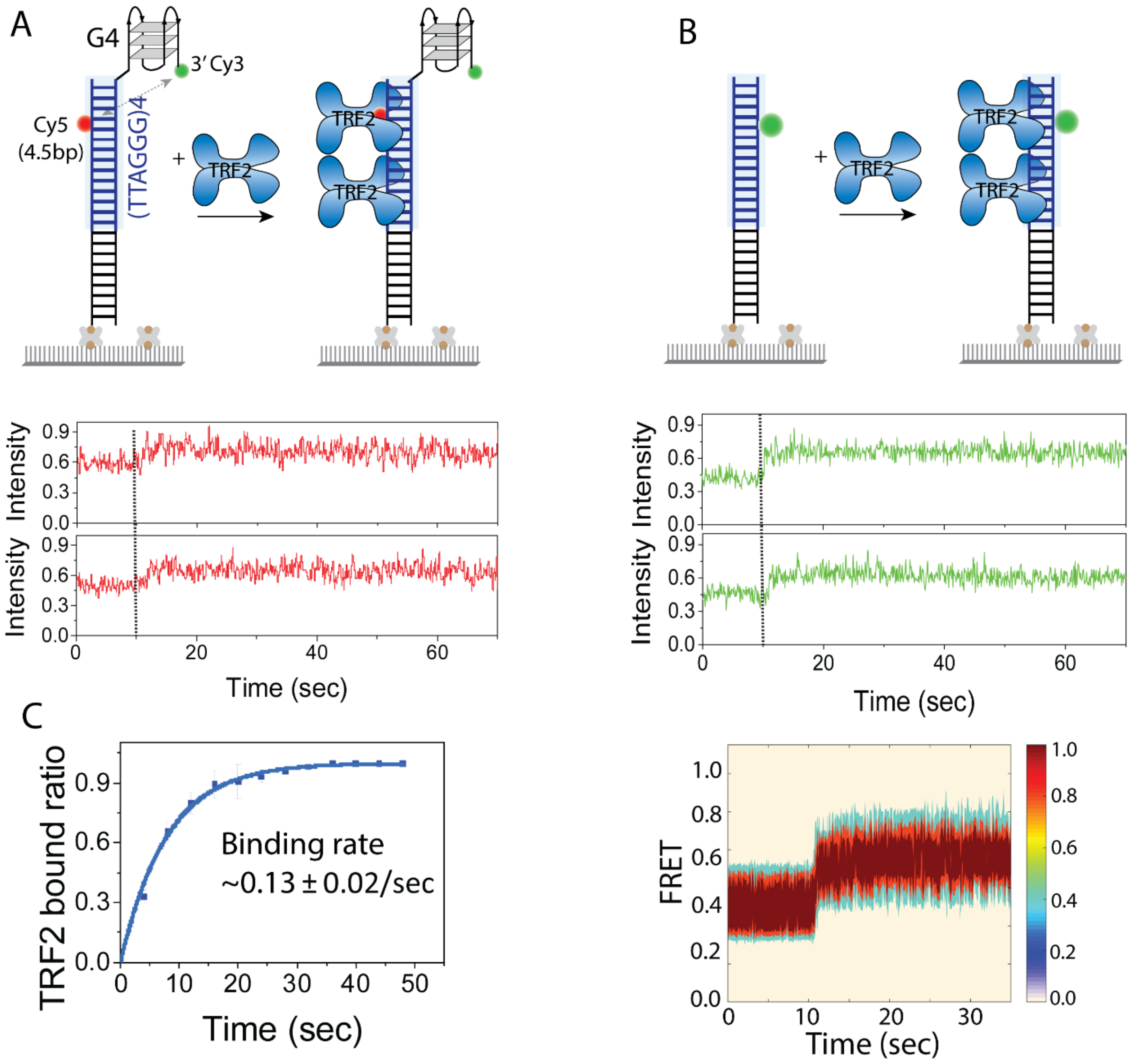

Figure S14. Protein induced fluorescence enhancement (PIFE) traces of TRF2 binding to telomeric duplex by applying red **(A)** and green **(B)** laser respectively. Heatmap of green PIFE traces. Dash line represent the time of TRF2 flow. PIFE assay is ideally suited for studying protein binding which does not result in FRET change. **(C)** Binding kinetics of TRF2 binding to telomeric duplex deduced by fitting the dwell time analysis of TRF2 binding collected from over 200 single-molecule PIFE traces.

SUPPLEMENTARY FIGURE 15

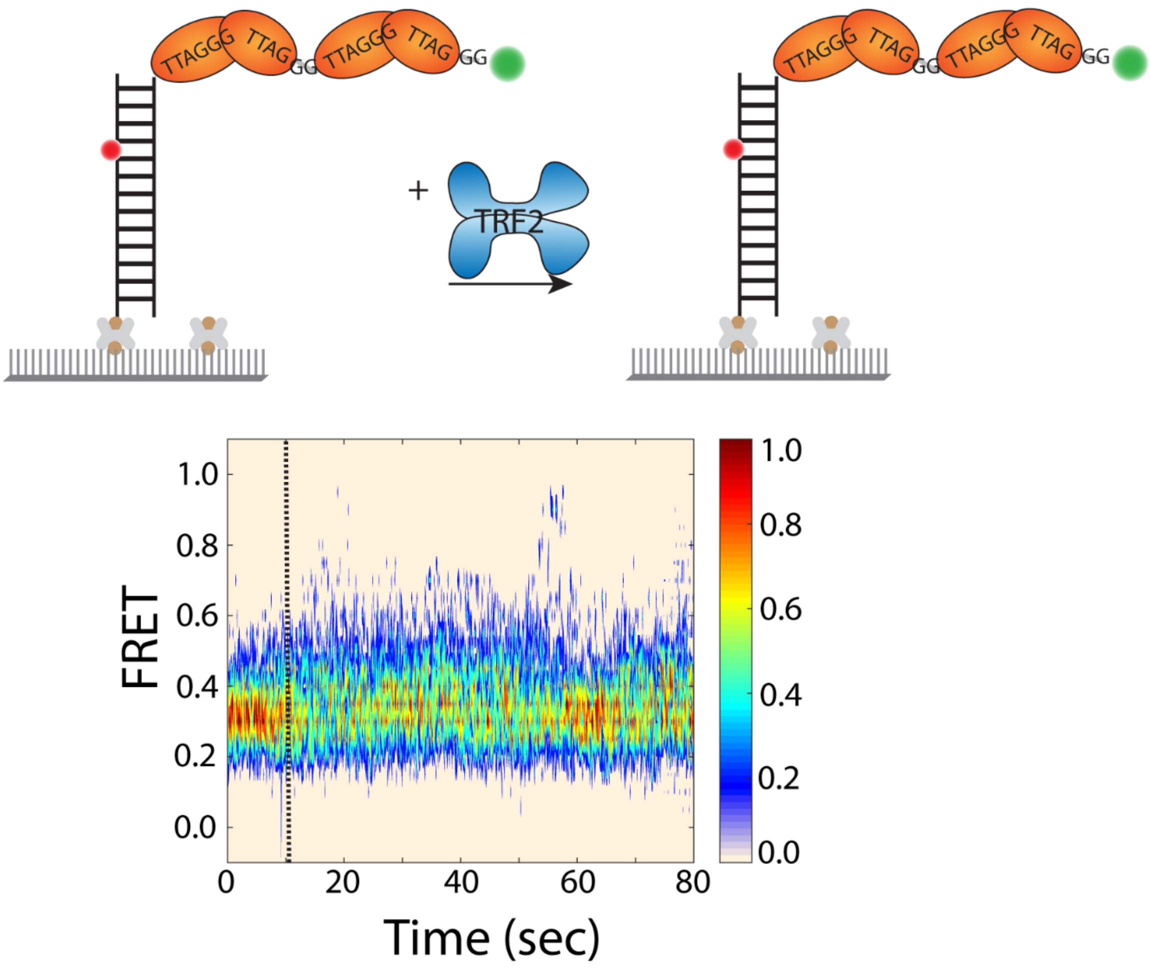

Figure S15. Control experiment in which TRF2 was applied to POT1 bound G4/4R overhang containing non-telomeric duplex. As expected, FRET or PIFE signals do not change due to no binding of TRF2 to non-telomeric duplex, hence no change in the FRET heat map based on over one hundred single-molecule traces.

SUPPLEMENTARY FIGURE 16

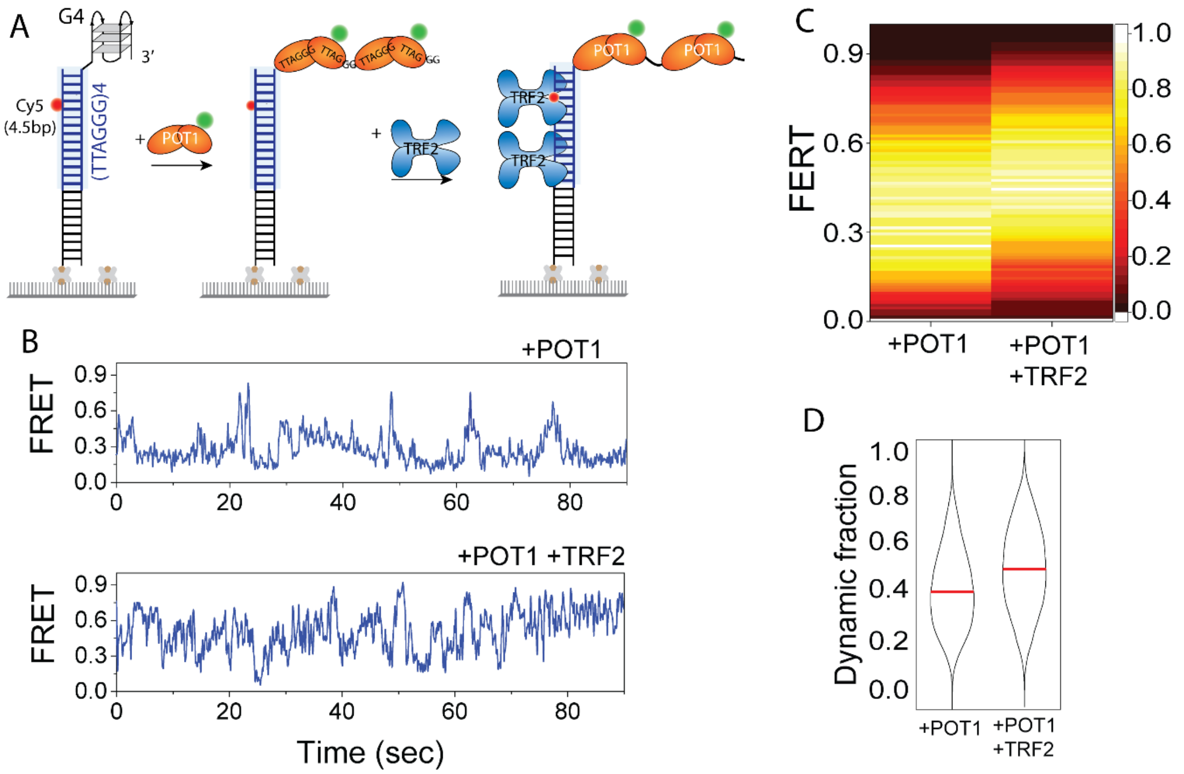

Figure S16. (A) Schematic model of Cy3-labeled POT1 binding to G4/4R overhang followed by TRF2 engaging with four TTAGGG repeats of telomeric duplex. **(B)** Representative single-molecule traces of POT1 alone (top) and with TRF2 (bottom). (**C & D**) The heatmap histogram **(C)** and violin plot **(D)** of POT1 alone and with TRF2. Consistent with the unlabeled POT1 and TRF2 experiment, we detect higher level of FRET fluctuation dynamics in the presence of TRF2.

SUPPLEMENTARY FIGURE 17

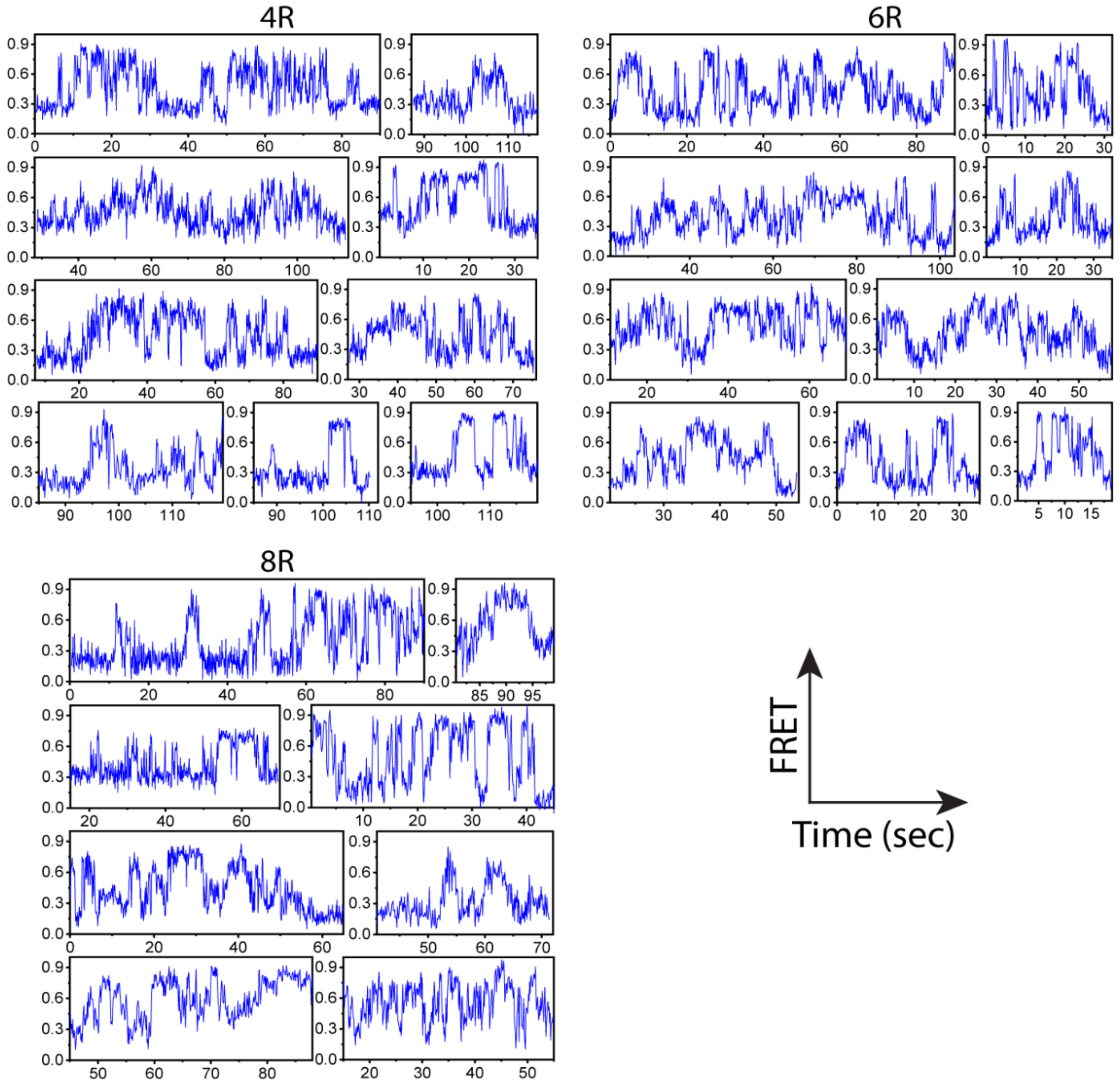

Figure S17. More representative single-molecule FRET traces of TRF2 bound to four TTAGGG repeats of telomeric duplex containing POT1 bound to 4R, 6R and 8R overhang respectively. These traces display that the same degree of dynamics persist regardless of the overhang length which ranges from 4R (24 nt) to 8R (48 nt), suggesting a highly conserved mode of movement.

SUPPLEMENTARY FIGURE 18

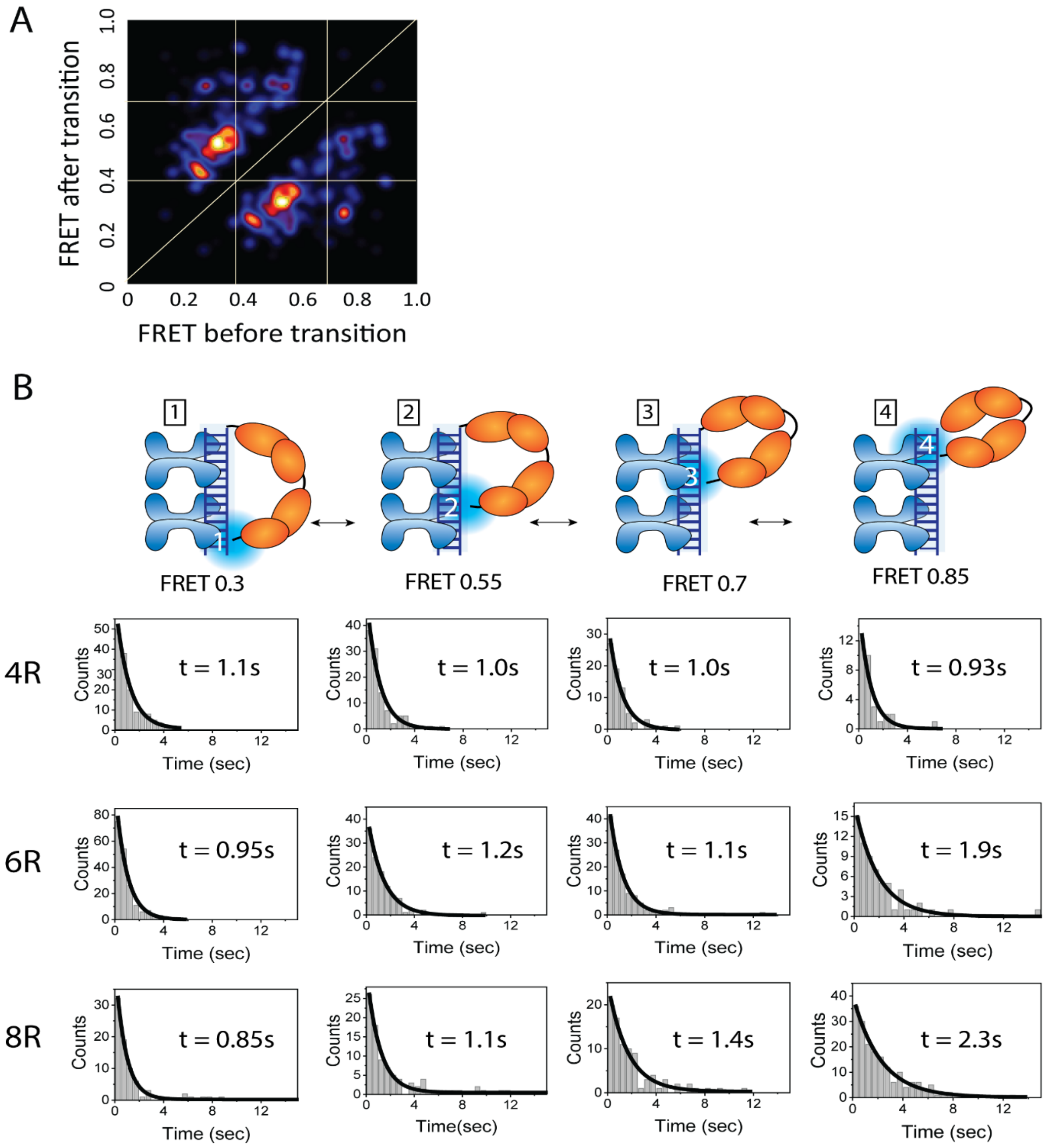

Figure S18. (A) Transition density plot (TDP) of POT1 bound G4/4R overhang containing telomeric duplex, demonstrating that the dynamic pattern of FRET fluctuation does not depend on the presence of telomeric DNA in the duplex, rather this pattern is inherent to POT1-overhang complex, **(B)** The proposed model of POT1 bound 4R telomeric overhangs moving up and down the TRF2 bound four repeats of TTAGGG containing duplex (Top). The dwell time of different dynamic FRET states (~0.35, ~0.55, ~0.7, ~0.85) of 4R, 6R and 8R overhang bound POT1 with TRF2 bound at the telomeric duplex (below). The individual lifetime of the different FRET state reveals the lifetime of highest FRET state (~0.85) was the highest in 8R followed by 6R and 4R, suggesting that the most looped state is stabilized by the longer POT1 bound overhangs, likely due to less tension generated in looping longer POT1-overhang complex.

SUPPLEMENTARY FIGURE 19

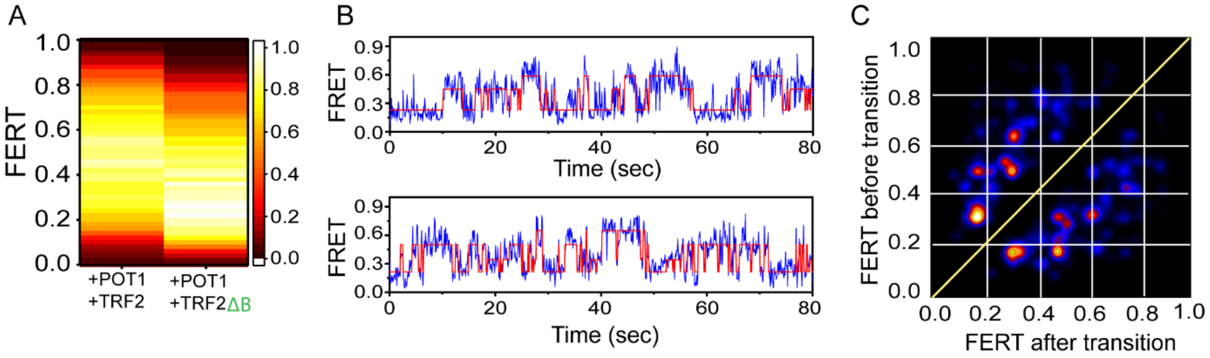

Figure S19. POT1 bound 8R overhangs does not reach high level of FRET when TRF2ΔB (TRF2 missing a basic domain) is bound to telomeric duplex, consistent with the role of basic domain in stabilizing the TRF2 binding to ds/ss junction. **(A)** FRET heatmap histograms POT1 bound 8R overhangs containing telomeric duplex bound with either TRF2 (left) or TRF2ΔB (right). **(B)** Representative smFRET traces (blue) fitted by HMM (red) of POT1 and TRF2ΔB bound. **(C)** Transition density plot (TDP) of both POT1 and TRF2ΔB bound.

SUPPLEMENTARY FIGURE 20

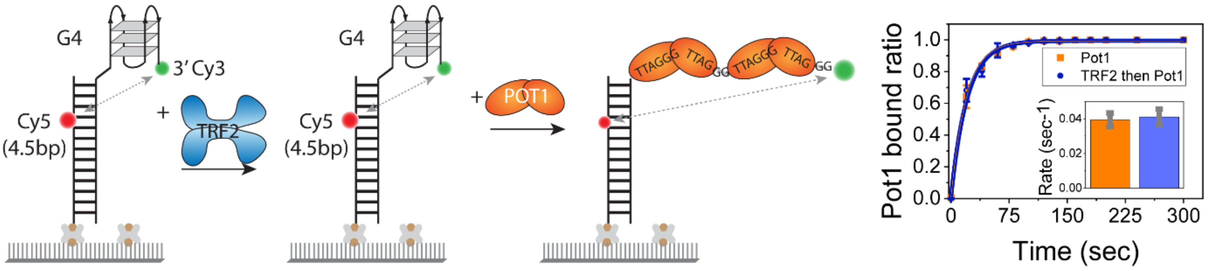

Figure S20. Binding kinetics of POT1 alone and TRF2 followed by POT1 addition to the non-telomeric duplex. The same rate found in both conditions simply confirms that TRF2 is not binding to non-telomeric duplex, hence the same binding kinetic of POT1 on overhang.
